# Evolutionary analysis of selective constraints identifies ameloblastin (AMBN) as a potential candidate for amelogenesis imperfecta

**DOI:** 10.1186/s12862-015-0431-0

**Published:** 2015-07-30

**Authors:** Frédéric Delsuc, Barbara Gasse, Jean-Yves Sire

**Affiliations:** Institut des Sciences de l’Evolution, UMR 5554, CNRS, IRD, EPHE, Université de Montpellier, Montpellier, France; Université Pierre et Marie Curie, UMR 7138 Evolution Paris-Seine, Paris, France

**Keywords:** Ameloblastin, Evolution, Enamel, Mammals, Purifying selection, Positive selection, Phylomedicine

## Abstract

**Background:**

Ameloblastin (AMBN) is a phosphorylated, proline/glutamine-rich protein secreted during enamel formation. Previous studies have revealed that this enamel matrix protein was present early in vertebrate evolution and certainly plays important roles during enamel formation although its precise functions remain unclear. We performed evolutionary analyses of AMBN in order to (i) identify residues and motifs important for the protein function, (ii) predict mutations responsible for genetic diseases, and (iii) understand its molecular evolution in mammals.

**Results:**

In silico searches retrieved 56 complete sequences in public databases that were aligned and analyzed computationally. We showed that *AMBN* is globally evolving under moderate purifying selection in mammals and contains a strong phylogenetic signal. In addition, our analyses revealed codons evolving under significant positive selection. Evidence for positive selection acting on AMBN was observed in catarrhine primates and the aye-aye. We also found that (i) an additional translation initiation site was recruited in the ancestral placental *AMBN*, (ii) a short exon was duplicated several times in various species including catarrhine primates, and (iii) several polyadenylation sites are present.

**Conclusions:**

AMBN possesses many positions, which have been subjected to strong selective pressure for 200 million years. These positions correspond to several cleavage sites and hydroxylated, O-glycosylated, and phosphorylated residues. We predict that these conserved positions would be potentially responsible for enamel disorder if substituted. Some motifs that were previously identified as potentially important functionally were confirmed, and we found two, highly conserved, new motifs, the function of which should be tested in the near future. This study illustrates the power of evolutionary analyses for characterizing the functional constraints acting on proteins with yet uncharacterized structure.

**Electronic supplementary material:**

The online version of this article (doi:10.1186/s12862-015-0431-0) contains supplementary material, which is available to authorized users.

## Background

Ameloblastin (AMBN), amelogenin (AMEL), enamelin (ENAM), amelotin (AMTN), odontogenic, ameloblast associated (ODAM) and SCPP-PQ1, are ameloblast-secreted proteins involved in various steps of the organization and mineralization of the enamel matrix of mammalian teeth. They belong to the large secretory calcium-binding phosphoprotein (SCPP) family (23 members known in humans), which appeared more than 450 million years ago (Ma) then diversified through gene duplication during vertebrate evolution [1–4]. In mammals, non-AMEL proteins account only for 10–20 % of the total enamel proteins, among which AMBN, also known as sheathlin [5, 6] or amelin [7], is the most abundant [8–10]. *AMBN* mRNA was sequenced in pigs [6], mice [11], humans [12, 13], cattles and guinea-pigs (direct submission to NCBI database). In non-mammals, *AMBN* cDNA was published only in a crocodile [14] and in the clawed toad [15]. Genomic DNA (gDNA) sequences are known in a lizard [16] and in the coelacanth [17]. These findings indicate that *AMBN* was present early during vertebrate evolution, and at least in the last common ancestor of sarcopterygians (420 million years ago, Ma [18]). However, the large evolutionary distance among the few AMBN sequences available in non mammalian tetrapods does not allow accurate comparison and further analysis, including new data in non mammalian sarcopterygians are needed to better understand AMBN evolution through such a large geological period.

AMBN is an intrinsically unstructured, enamel matrix protein (EMP), and its functions remain unclear. It has been suggested either to be a structural component of the enamel matrix playing a role in maintaining the prismatic structure of growing enamel crystals at the rod and interrod boundaries [6, 19, 20], or to be involved in ameloblast adhesion [21–23], or to function as a growth factor [24–27] or as a signaling molecule [26, 28]. AMBN possesses two calcium-binding domains that interact with Ca^2+^ ions after being liberated by proteolysis [29–31]. The transcription start site (TSS) and the promoter region of *AMBN* were analyzed in the mouse [32]. This region contains cis-acting elements that function both to enhance and suppress transcription, some of them regulating transcription activity in mesenchymal cells *in vitro* [33]. *Cbfa1*, the essential transcription factor for osteoblast differentiation, was shown to play an important role in *AMBN* transcription [34].

It is worth noting that (i) *AMBN* expression was reported during craniofacial bone development in rats [35], and (ii) *in vitro* and *in vivo* experiments have suggested that this protein could also play a role in bone formation and repair [27, 36, 37]. These findings could indicate that AMBN plays a role in early bone formation and modeling, but they have been contradicted by a study showing no implication in bone modeling and repair [38]. Moreover, a possible function in bone development remains elusive as (i) *AMBN*^−/−^ mice do not exhibit apparent bone phenotype [21], (ii) *AMBN* is subjected to pseudogenization in birds and in mammalian species, in which the capability to form either enamel or teeth has been lost, e.g., xenarthrans, pangolins and baleen whales, indicating that AMBN is a tooth specific protein [39–41], and (iii) *AMBN* expression was found to be restricted to teeth in rodents and in a crocodile [9, 14]. Finally, *AMBN*^−^/^−^ mice develop severe enamel hypoplasia only, a phenotype that indicates a crucial role of AMBN in enamel formation [21].

Because of its important role in mammalian enamel formation, for long *AMBN* has been considered a candidate gene for amelogenesis imperfecta hereditary type 2 (AIH2), a human genetic disease [42–46]. In humans, *AMBN* is located on the long arm of chromosome 4 (4q13-21), containing the gene locus for the autosomal dominant hypoplastic form of AIH2, that affects enamel formation and is the most prevalent amelogenesis imperfecta (AI) type (85 % of all inherited AI) [12, 47]. However, it is only recently that the first case of a disease-associated mutation of *AMBN* (homozygous exon 6 deletion) was reported in a family having hypoplastic AI [48]. The lack of other cases of *AMBN*-associated AIH2 strongly contrasts with the important role played by AMBN during amelogenesis. This contradiction could be explained by an autosomal recessive pattern of inheritance as demonstrated in *AMBN*^+/−^ mice that do not exhibit an apparent dental phenotype [49].

Interestingly, human ameloblastoma, a benign but destructive tumor, expresses *AMBN* transcripts with tumor-specific mutations [13, 50, 51]. Such expression suggests that *AMBN* plays a role in epithelial odontogenic tumors. *AMBN* is also expressed in osteosarcoma cells [27]. In humans and some mammals, amyloidosis has been associated with calcifying epithelial odontogenic tumors (CEOT) and *AMBN* was found to be highly expressed in these cells [52]. *AMBN* deficiency was proposed to be the cause of the odontogenic tumors seen in 20 % of the *AMBN*^−^/^−^ mice [21]. Also, tumor suppressor genes *P21* and *P27* are upregulated in *in vitro* transfected ameloblastoma cells [22].

In recent years, a series of detailed evolutionary analyses of mammalian SCPPs have been conducted (AMEL [53]; ENAM [54]; MEPE [55]; AMTN [56]; DMP1 [57]) with the aim to (i) reveal regions and residues important for the protein function, (ii) predict or validate mutations responsible for genetic diseases, and (iii) understand their mode of evolution, origin and relationships [3, 4]. Here, we perform such an evolutionary analysis of mammalian AMBN sequences in order to predict functionally important sites of the protein and to identify candidate disease-associated mutations responsible for AI in human, an approach called phylomedicine [58].

## Material and methods

### AMBN sequences and alignment

A total of 56 mammalian full-length *AMBN* coding sequences, representative of the main lineages, were extracted from the NCBI [59] and Ensembl [60] databases: four published, full-length sequences (human, pig, mouse and rat); two sequences published only in GenBank (cow and guinea-pig), all including the UTRs; 33 computer-predicted sequences, i.e., available from the automatic analysis of sequenced mammalian genomes; and 17 sequences obtained using BLAST from whole genome shotgun data.

The *AMBN* sequences of armadillo and sloth (Xenarthra), and of aardvark (Afrotheria) are particular because the former is enamel-less in adult and the two others lack enamel, even in juveniles [41, 61]. AMBN being a tooth-specific protein [39, 40] the gene has accumulated mutations after the ability to form enamel was lost, or reduced, in these lineages [41]. Therefore, we performed our evolutionary analyses with two datasets: the complete dataset of 56 sequences and a reduced dataset of 53 functional mammalian AMBN sequences. The published crocodilian sequence (*Caiman crocodilus*) was also used as outgroup for calculation of the mammalian ancestral sequence. Species names and sequence references are indicated in the Additional file 1.

The coding regions of the *AMBN* sequences were translated into putative amino acid sequences, aligned to the human sequence using Clustal X 2.0 [62], and checked by hand using Se-Al v.2.0a11 software [63, 64]. Each of the computer-predicted sequences was carefully checked against the published sequences, taking into account the intron/exon boundaries. When necessary, they were completed and/or corrected using (i) Blast search against the Ensembl genome database, (ii) the NCBI trace archives, (iii) the whole genome shotgun (WGS) repository sequences, and (iv) resequencing by performing classical PCR on DNA extracted from alcohol-preserved tissues. In our final alignment only a few residues were missing, representing less than 1 % of the data. The positions with missing data were included in our analyses and treated as “unknown states”.

### 5' untranslated region (5' UTR) and signal peptides

Taking the published mRNA sequences as templates, we obtained the putative 5' UTR (i.e., exon 1 and beginning of exon 2) of *AMBN* sequences in 49 genomes using BLAST. Then, using Clustal X 2.0 and Se-Al v.2.0a11, we aligned these sequences against the published mammalian sequences of exons 1 and 2 with particular attention paid on the exon-intron boundaries. The transcription start site (5' end of exon 1) was defined by default, but this region was not useful for our analysis. The 49 sequences were analyzed to identify the ATGs that could be correct translation initiation sites (TIS). We used the DNA functional site miner [65] that predicts functional TIS i.e., possessing the highest Kozak consensus score [66, 67]. All identified putative signal peptides (SPs) were analyzed using the Signal P 3.0 server [68]. This software predicts the location of the three characteristic regions (n-, h- and c- regions) of a SP, the putative cleavage site of the protein, and calculates the probability for each SP to be functional [69].

### Phylogenetic reconstruction

The 53 functional mammalian *AMBN* sequences were aligned based on their amino acid translations using MAFFT [70] within Geneious R6.03 [71]. The alignment was then restricted to the length of the human AMBN sequence by excluding all sites containing gaps in non-human sequences and the final stop codon. This procedure resulted in a dataset containing 1341 nucleotide sites (447 codons/amino acids versus 500 when including gaps). Then the three non-functional sequences (aardvark, armadillo and sloth) were added to this dataset using the program MACSE specifically designed to deal with frameshifts and stop codons [72]. Ambiguously aligned codons were then excluded using Gblocks [73] leading to a dataset with 1239 nucleotide sites (413 codons) for 56 taxa. Maximum likelihood (ML) phylogenetic reconstructions were performed on the two nucleotide alignments under the GTR + GAMMA model using RAxML 7.3.2 [74]. The statistical support for the ML tree was evaluated using 100 bootstrap replications of the initial ML search. The platypus (*Ornithorhynchus anatinus*) sequence was used as outgroup in all analyses.

### Selection analyses

We evaluated the selective constraints acting on AMBN by performing a number of statistical tests based on the ratio of non-synonymous (dN) to synonymous (dS) substitutions (dN/dS or ω) using the codeml program of the PAML 4.7a package [75]. In all analyses, we used the mammalian species tree topology as inferred by Meredith et al. [76], except for the position of the placental root, which follows Romiguier et al. [77]. We first tested for significant among-site variation in dN/dS ratio along the AMBN molecule by using site-specific codon models for the detection of positive selection. This was achieved by comparing the fit of a nearly neutral model (M7) versus a model (M8) that allows a fraction of positively selected sites [78, 79]. These two models were compared using a hierarchical likelihood ratio test (LRT) [80]. A conservation index (CI) was then calculated for each codon from the Bayes Empirical Bayes (BEB) estimated mean values of ω obtained under the M8 model following the procedure proposed by Burk-Herrick et al. [81]. The CI was defined as 1-ω with values of neutral (ω = 1) and positively selected sites (ω > 1) being set to 0 in order to graphically represent site-specific selective constraints along the AMBN molecule [82].

The branch specific variation of dN/dS in AMBN across the mammalian species tree was jointly reconstructed from the complete 56-taxa dataset with divergence times while controlling the effect of life-history traits [83] using the Bayesian framework implemented in the CoEvol program [84]. The dN/dS ratio was estimated under the dsom procedure together with the 24 calibration constraints issued from Benton et al. [85] that were compatible with our taxon sampling to estimate divergence times. The prior on the root node was set to 220 Ma with a standard deviation of 60 Ma following the expert estimate for the split between Monotremata and Placentalia reported on the TimeTree2 website [86]. The values of the three life-history traits incorporated into the analysis (body mass, longevity, and sexual maturity) were extracted from the PanTheria database [87]. Two independent MCMC were run for a total of 2500 cycles sampling points every cycle. The first 500 points of each MCMC were then excluded as the burnin, and inferences were made from the remaining 2000 sampled points.

To further test for significant variations in dN/dS along specific branches, we used the branch model implemented in codeml allowing ω to vary among specific branches and/or clades [88]. Hierarchical LRTs were calculated between the one-ratio model (M0) and alternative models (M2ω, M4ω, M5ω, M8ω), in which branches with a mean dN/dS > 0.6 as estimated in the previous Bayesian reconstruction were allowed to have their own ω (see Table 1).

**Table 1:** Results of likelihood ratio tests for positive selection in AMBN

Models	Hypotheses		LRT		
	Null hypothesis	Alternative hypothesis	−2ΔLnL (df)	*p*-value	Sites or branches
Site Models					Sites under M8 (BEB)
53-taxa data set	M7: beta	M8: beta&ω	42.77 (2)	*p* < 0.001	135 (ω = 1.49 ± 0.06; PP = 0.99)
					328 (ω = 1.50 ± 0.04; PP = 1.00)
					360 (ω = 1.48 ± 0.12; PP = 0.96)
					375 (ω = 1.49 ± 0.06; PP = 0.99)
					394 (ω = 1.48 ± 0.11; PP = 0.97)
Branch Models					Branches
53-taxa data set	M0: One-ratio model	M2ω: 2-ratio model	29.89 (1)	*p* < 0.001	Background (ω = 0.44), Catarrhines (ω = 1.32)
	M0: One-ratio model	M5ω: 5-ratio model	52.16 (4)	*p* < 0.001	Background (ω = 0.43), Catarrhines (ω = 1.32), Elephant (ω = 0.73), Elephant shrew (ω = 0.75), Aye aye (ω = 1.13)
56-taxa data set	M0: One-ratio model	M2ω: 2-ratio model	18.11 (1)	*p* < 0.001	Background (ω = 0.45), Catarrhines (ω = 1.11)
	M0: One-ratio model	M4ω: 4-ratio model	19.73 (3)	*p* < 0.001	Background (ω = 0.45), Armadillo (ω = 0.67), Sloth (ω = 1.10), Aardvark (ω = 0.76)
	M0: One-ratio model	M5ω: 5-ratio model	39.62 (4)	*p* < 0.001	Background (ω = 0.44), Catarrhines (ω = 1.11), Armadillo (ω = 0.67), Sloth (ω = 1.10), Aardvark (ω = 0.76)
	M0: One-ratio model	M8ω: 8-ratio model	58.14 (7)	*p* < 0.001	Background (ω = 0.42), Catarrhines (ω = 1.11), Armadillo (ω = 0.68), Sloth (ω = 1.10), Aardvark (ω = 0.76), Elephant (ω = 0.68), Elephant shrew (ω = 0.76), Aye aye (ω = 1.07)

*BEB* Bayes Empirical Bayes, *PP* Posterior Probability

### Ancestral sequence reconstruction

Ancestral sequence reconstruction of mammalian *AMBN* was computed with FastML [89] on the FastML server [90]. The *AMBN* sequence of the caiman (*Caiman crocodilus*) was added to the 53-taxa alignment using MAFFT in order to serve as outgroup. FastML was used for inferring ancestral amino acid sequences at each node of the nucleotide-derived ML tree under the LG + G8 + F model using the marginal reconstruction procedure including indels.

## Results

**Fig. 1:**
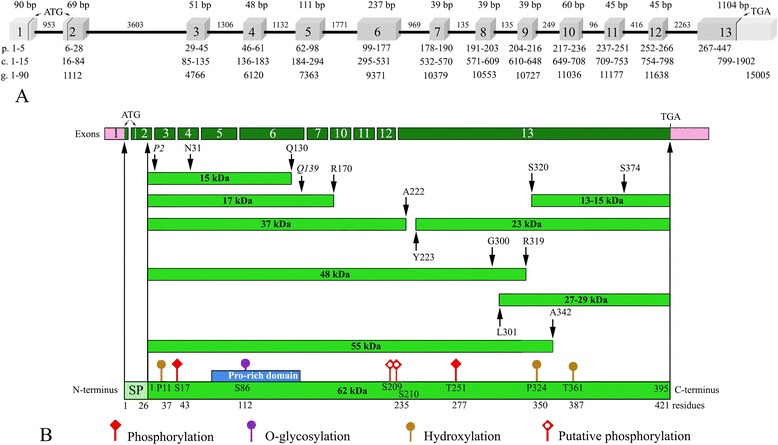
Gene structure and proteolytic cleavage products of ameloblastin. **a**: Human *AMBN*. Gene structure based on the reference gene sequence (Genbank accession No NC_000004.11). Exons are numbered from 1 to 13. Two translation initiation sites (ATG) are found: in exon 1 and exon 2. The untranslated regions are in light grey. The number of base pairs (bp) is indicated above each exon. The nomenclatural counts of encoded amino acids (p.), cDNA bp (c.) and gDNA bp (g.) are also indicated below each exon. Introns are symbolized by the black lines (not at scale) with the nucleotide number indicated above. The stop codon (TGA) is located in exon 13. **b**: Porcine AMBN. Diagram showing the cleavage products and their corresponding location on the protein sequence (after [6, 20, 29, 99, 101, 116]). Top: *AMBN* exons. Note that exons 8 and 9, which encode 26 residues in humans are lacking in porcine *AMBN*. Bottom: AMBN protein including either the 26 residues forming the signal peptide-SP (1–421) or not (1–395). Several remarkable residues were identified: two phosphorylated residues (S^17^, T^251^) and two putatively phosphorylated residues (S^209^, S^210^), two O-glycosylated residues (S^86^, T^361^), two hydroxylated prolines (P^11^, P^324^), and the proline-rich region. The secreted protein has an apparent molecular weight of 62 kDa; cleavage sites leading to various AMBN fragments found *in vivo* are indicated in roman characters, two MMP20 cleavage sites found in *in vitro* studies are in italics

For a better understanding of the results presented below, our current knowledge of *AMBN* structure (exemplified in humans) and the location of cleavage sites of the protein (identified in pigs) are illustrated in Fig. 1.

### Alignment and sequence comparisons

The 56 AMBN sequences studied here represent 46 families distributed in 19 orders and are representative of the current mammalian phylogenetic diversity (Additional file 1). The alignment of the 53 functional sequences resulted in a 500 amino acid long sequence, when considering methionine in exon 1 (Additional file 2), and including gaps required for accurate alignment. For convenience of presentation, the duplicated exons 9c, 9e, and 9d were not shown: they were nearly identical and only found in a few species (see below).

Sequence comparison revealed a few insertions, the largest being related to the duplication of small exons in 12 species (see below). These additional exons account for a large part of the length variation observed among the sequences (Additional file 2). In the 10 rodent AMBN analyzed, an insertion of 4 or 5 residues (QG/NMAP) was identified in the region encoded by exon 3. This event likely occurred in the rodent ancestral branch after the divergence with lagomorphs (rabbit and pika), which do not possess this insertion. The insertion is located close to a remarkably conserved region and probably results from the duplication of the neighboring PGMAS residues (Additional file 2). In hedgehog, six residues were inserted in the region encoded by exon 13 at positions 365–370, and in marsupials three amino acids (IKQ) were inserted in the region encoded by the 3′ end of exon 2. In addition, a few insertions and deletions (indels) of one to five residues were found distributed in the whole sequences and no sequence repeats were identified, to the exception of the exon duplication reported below.

### Additional exons

Human *AMBN* possesses two exons, exons 8 and 9, which are absent in rodent sequences. Therefore, we screened the DNA region located between exons 7 and 10 in the mammalian genomic sequences to check whether additional exons were present in this region. We show that a gene structure composed of 11 exons (i.e., exons 1 to 7 and 10 to 13) characterizes 41 mammalian *AMBN* out of the 53 functional sequences investigated. This structure is also conserved in caiman, which indicates that the ancestral mammalian *AMBN* most probably possessed 11 exons. In 12 species, however, *AMBN* displays additional exons in this particular region. They are eight simiiform primates (out of nine), three laurasiatherians (horse, microbat and hedgehog), and one afrotherian (elephant shrew) (Additional file 2): one exon in orangutan and microbat; two in humans, chimpanzee, gibbon, squirrel monkey, marmoset and hedgehog; three in elephant shrew; four in baboon and macaque; and six in horse. These additional exons have the same length (39 bp) and are nearly identical in sequence to exon 7 (Additional file 2). These features strongly suggest that these exons originated all from tandem duplications of a short DNA region containing exon 7. This region is the only *AMBN* location, in which additional exons were identified. The two exons found between exons 7 and 10 in human *AMBN* were numbered 8 and 9a; therefore, the extra exons found between exon 9a and 10 were numbered 9b, 9c, etc. In the horse, the last duplicated exon was numbered 9e. It is worth noting that closely related species such as shrew *vs.* hedgehog, megabat *vs.* microbat, etc. lacked duplicated exons, a finding which strongly suggests that the duplications occurred independently within the different lineages (Additional file 2). However, in primates, duplication of this short DNA region is found only in the Simiiformes (Platyrrhini + Catarrhini), which could indicate that the first duplication likely occurred in the common ancestor of simiiforms, after this lineage diverged from Tarsiiformes, and was subsequently conserved in all simiiform lineages except in gorilla. The presence of these duplicated exons contributes to a substantial increase in length of the encoded protein: from 421 amino acids (aa) on average in the majority of sequences to e.g., 447 aa in humans, 473 in baboon, and 499 aa in horse, which is the largest AMBN mammalian sequence known to date (Additional file 2). These exons also encode two to four prolines, which contribute to enlarge the proline-rich region of the protein.

Analyses of exon/intron boundaries in representative species indicated that the 21 nucleotides on each side of the exons are rather well conserved (Additional file 3). The unchanged nucleotides in these intronic regions could be important for intron splicing.

### Translation initiation site (TIS) and signal peptide (SP) analyses

**Fig. 2:**
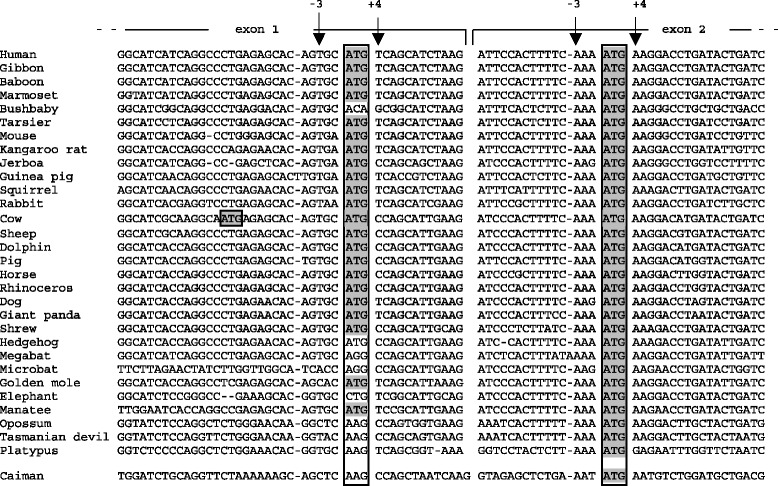
Location of putative translation initiation sites (TIS) in 30 mammalian and one crocodilian *AMBN* sequences. The arrows (−3, +4) point to nucleotide positions that play an essential role in the initiation of the translation. Two putatively functional TIS (squared ATGs on grey background in exons 1 and 2) are present in placental sequences, but a few exceptions, while only one TIS is identified in marsupials, platypus and crocodile. An additional, putatively functional TIS is present in cow *AMBN*

Alignment of the 5' region of the *AMBN* sequences revealed that the 3' end of exon 1 and the 5' end of exon 2 are well conserved (Fig. 2). Depending on the species, in exon 1, exon 2 and/or in both, DNAFSMiner identified one to five ATGs in the right context i.e., that does not lead to in-frame downstream stop codons. For each sequence analyzed, the first ATG was always predicted as being the functional TIS. In most placentals the putative TIS was located in exon 1, but with a few exceptions (bushbaby, hedgehog, bats and elephant), in which it is found in exon 2 as in marsupials, and monotremes (Fig. 2). In placentals, the TIS was located at the same position in the 3' region of exon 1, with the exception of Bovinae*,* in which another TIS, was identified upstream. In humans, for instance, DNAFSMiner predicted that the first ATG (starting at position 76, exon 1) is the correct functional TIS (score = 0.844). However, when exon 1 was not included in the analysis, the first ATG in exon 2 (position 106) was also predicted as the functional TIS (score = 0.627). This means that the second TIS could be used if that encoded by exon 1 was lacking, i.e. by using different promoter and transcription initiation sites.

Interestingly, the predicted TIS in exon 1 exhibits a weak Kozak consensus as it lacks a purine (A/G) in position −3 while possessing a pyrimidine in position +4. However, such ATG could serve as the initiator codon [66]. In contrast, the ATG located in exon 2 has a strong Kozak consensus (purines in −3 and +4). As a consequence, translation could occur not only at the first (weak) TIS but also at the next (strong) TIS, resulting in the synthesis of two proteins [66]. The additional TIS identified in cow *AMBN* exon 1 displays also a strong Kozak consensus (a purine at positions −3 and +4), and DNAFSMiner predicted this TIS as the functional one. This additional TIS is also present in the water buffalo *Bubalus bubalis* and the American bison *Bison bison* (Bovinae) but is absent in the sheep, goat and Tibetan antelope *Pantholops hodgsonii* (Antilopinae) *AMBN* sequences (not shown), which suggests that the CTG to ATG substitution occurred along the Bovinae lineage after its divergence from Antilopinae some 20 Mya [91].

Our alignment indicates that the TIS in exon 2 is conserved in all *AMBN* analyzed, including that of the caiman, which means an ancient origin for this ATG. In contrast, the ATG in exon 1 was probably recruited in the eutherian ancestor, after an A to T substitution changed the ancestral AAG to ATG (Fig. 2). This ATG appears to have been secondarily lost independently in various lineages or species: ATG to CTG in elephant*,* ATG to AGG in bats, and ATG to ACA in bushbaby. However, in most primates, glires, most laurasiatherians, and five afrotherians out of six studied, the translated, putative AMBN sequences exhibit two methionines: the methionine encoded by the ATG in exon 1, numbered M^1^, and the one encoded by the ATG in exon 2, M^11^. In cow the first methionine is located five residues upstream M^1^.

The analysis of the putative SPs with SignalP revealed a similar, high probability for the SP starting either at M^1^ or M^11^. As a consequence, the SP of most placental AMBN can be either large (26 aa), when starting at M^1^, or short (16 aa) when beginning at M^11^. The three typical regions — positively charged n-region, hydrophobic h-region, and polar c-region — were found in both SPs (Additional file 4). The maximal cleavage site probability was always located between Ala^26^ and Val^27^. The corresponding predicted cleavage site is strongly supported by the presence of these two residues in all AMBN sequences analyzed, excepted for the platypus, in which Val is replaced with Ile from the same amino acid group (Additional file 2).

### Polyadenylation sites (PS)

Several putative alternative polyadenylation signals (PS) were identified in the 3' UTR of the mammalian *AMBN* sequences (Table 2). This region is large, ranging from 441 to 557 bp in representative species of the main lineages (493 bp in the caiman). The number of PS ranged from two in the platypus to five in several species, including humans, and were numbered PS1 to PS5, from the stop codon onwards. PS were either the highly conserved hexamer AATAAA found at three locations (PS2, PS3, PS5) or the common variant ATTAAA found at two locations (PS1, PS4) (Table 2). In our dataset the latter was less conserved than the canonical signal, and it was not found in the caiman sequence. When both present, PS1 and PS2 are close to one another and can be considered a single PS; PS3 is found in all sequences but the platypus; PS4 is present in rodents and primates, in some laurasiatherians and in marsupials; PS5 is found in all sequences. The caiman *AMBN* possesses PS2, PS3 and PS5. In marsupials, PS4 and PS5 are close one to each other. Altogether these findings suggest that (i) three PS (PS2, PS3 and PS5) were present in the 3'UTR of the last common ancestral mammalian *AMBN* and (ii) PS3 was secondarily lost in the platypus lineage.

**Table 2:** Location of putative alternative polyadenylation signals (PS) in the 3' UTR of *AMBN* cDNA sequences

Species	Length	PS1	PS2	PS3	PS4	PS5
	3'UTR	ATTAAA	AATAAA	AATAAA	ATTAAA	AATAAA
Human	553	106-111	129-134	189-194	415-420	536-541
Bushbaby	483	77-83	100-105	157-162	344-349	466-471
Tree shrew	553	106-111	129-134	189-194	412-417	536-541
Mouse	543	-	127-132	184-189	403-408	526-531
Squirrel	539	105-110	128-133	188-193	406-411	521-526
Rabbit	549	106-111	129-134	189-194	410-415	532-537
Cow	555	106-111	129-134	189-194	419-424	538-543
Horse	556	106-111	129-134	189-194	-	539-544
Dog	557	107-112	130-135	190-195	-	540-545
Microbat	513	-	109-114	169-174	377-382	497-502
Elephant	556	-	126-131	186-191	-	537-542
Opossum	540	121-126	-	209-214	495-500	520-525
Platypus	441	116-121	-	-	-	426-431
Caiman	493	-	95-100	298-303	-	475-480

TGA-------------PS1---PS2--------PS3-----------------------PS4--------------PS5--AAAAAAA

The PS are given for 13 representative species of the main mammalian lineages and the crocodile. Bottom: schematical representation of PS locations on the human *AMBN* sequence from the stop codon (TGA) to the poly(A); an hyphen represents 10 nucleotides

### Amino acid composition

EMPs are characterized by their high amount of proline (P) and glutamine (Q), and are included in the P/Q-rich sub-family of SCPPs [92]. In the seven representative species analyzed for AMBN residue composition, including caiman, proline is well represented (14.09 % in average for the full-length sequence and 23.78 % in average for the proline rich region) compared to the 5.1 % encountered in most proteins (Additional file 5). Glutamine (8.7 and 15.7 %, respectively) is also well represented compared to the overall value of 4.0 %. It is worth noting that the percentage of proline in both the full-length sequence and the proline-rich domain is relatively similar from caiman to humans, while the glutamine percentage is decreasing from 11.79 to 6.49 % and from 20.93 to 11.20 %, respectively. These two amino acids represent an average of *ca* 23 % of the residues in the full-length AMBN sequence and *ca* 40 % in the proline-rich domain. In the ancestral mammalian AMBN (see below) these amino acids represent *ca* 24 % (*P* = 14.1; Q = 9.8 %) and *ca* 42 % (22.2 + 20.2), respectively (Additional file 5).

### Ancestral AMBN and enamel-less species

In addition to its slightly higher P/Q percentage compared to living species, the ancestral mammalian *AMBN*, calculated from the 53 functional *AMBN* and using the caiman sequence as outgroup, displays a structure composed of 11 exons (1–7; 10–13), i.e., without duplicated exons between exons 7 and 10. There is a single TIS located in exon 2. Otherwise, the ancestral AMBN sequence is structurally similar to that of most mammalian AMBN and, in particular, possesses all the important positions and domains reported above.

**Fig. 3:**
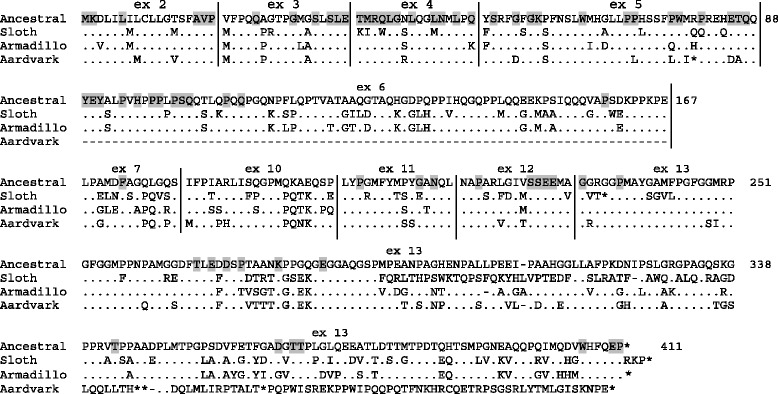
Alignment of AMBN sequences from three enamel-less mammals with the putative ancestral mammalian AMBN sequence. Exon limits are indicated by vertical lines. The residues conserved during mammalian evolution (see Fig. 5b) are on gray background. In the aardvark genome the region containing exon 6 is deleted. (.): residue identical to the ancestral AMBN residue; (−): indel; (=): unknown amino acid; *: stop codon

Sloth, armadillo and aardvark sequences were aligned with the ancestral AMBN. This alignment revealed that selective pressures on the AMBN sequence of these enamel-less species were more relaxed in sloth and aardvark than in armadillo (Fig. 3). Sloth and aardvark AMBN show (i) numerous residue substitutions, some having occurred in conserved positions, (ii) premature stop codons resulting from nucleotide substitution, (iii) frameshifts in the region encoded by the large exon 13 resulting from nucleotide insertion or deletion, and (iv) nucleotide substitutions in the splicing sites (not shown). In addition, a large deletion has occurred in the DNA region of aardvark *AMBN* between exons 5 and 7, and exon 6 has disappeared. Taken together these features indicate that *AMBN* is no longer functional in these two species and was subjected to pseudogenization. In contrast, armadillo *AMBN* does not exhibit stop codons and frameshifts, and residue substitutions are less numerous. However, several amino acid substitutions have occurred on conserved positions that were suggested above to play an important role for the protein function (Fig. 3). These data indicate that armadillo *AMBN* either was recently inactivated or is still active but the encoded protein is probably defective.

### Phylogenetic tree

**Fig. 4:**
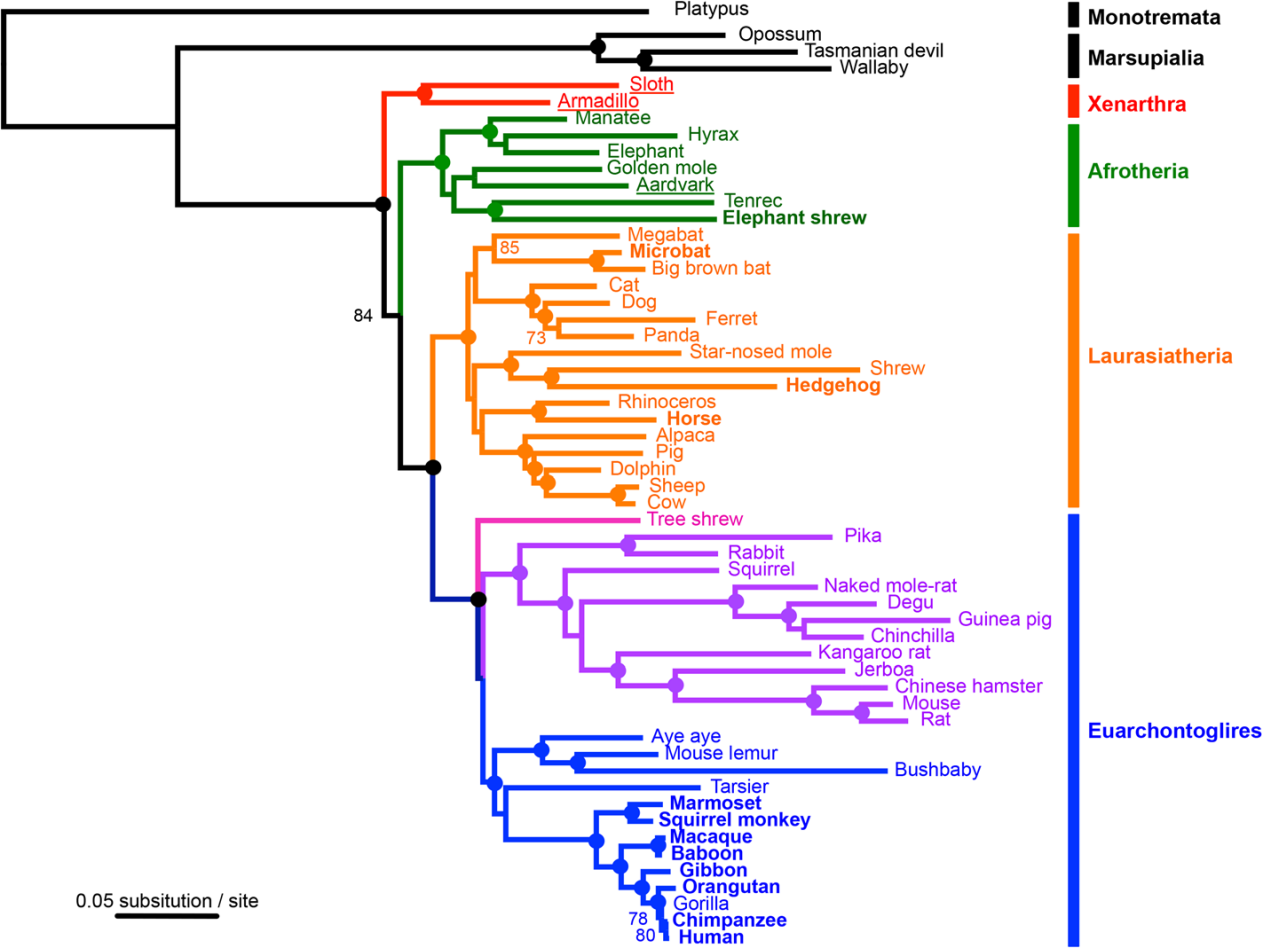
Maximum likelihood tree (GTR + GAMMA model) using the complete dataset of 56 mammalian *AMBN* sequences. The platypus (*Ornithorhynchus anatinus*) was used as outgroup. Bootstrap percentages are indicated. Nodes with more than 90 % bootstrap support are indicated with dots and nodes with no value received less than 70 % bootstrap support. Placentals are colored by main clades: Xenarthra (red), Afrotheria (green), Laurasiatheria (orange), Scandentia (pink), Glires (purple) and Primates (blue). Species possessing various copies of exon 7 are indicated in bold (see also Additional file 2). The three enamel-less species are underlined. Branch lengths are expressed as substitutions per site

The ML phylogram inferred from the complete 56-taxa nucleotide data set under the GTR + GAMMA model is presented in Fig. 4. This tree illustrates the strong phylogenetic signal contained in AMBN for resolving the phylogeny of mammals. Indeed, the ML topology supports the four major placental clades: Xenarthra, Afrotheria, Laurasiatheria and Euarchontoglires, and the grouping of the latter two in Boreoeutheria. Furthermore, almost all nodes are fully resolved and supported by bootstrap values of more than 90 % with the exception of, for instance, the relationships among laurasiatherian orders or the position of tree shrew within Euarchontoglires (Fig. 4). The ML estimate of the gamma shape parameter (α) was 0.92, which underlines the relative homogeneity of the site-specific substitution rates along the AMBN molecule. Among placentals, strong variations in substitution rates are revealed with contrasted branch lengths observed in the ML tree, with fast evolving taxa such as tenrec and elephant-shrew in Afrotheria, shrew and hedgehog in Laurasiatheria, all Glires except pika and squirrel, and bushbaby in Primates within Euarchontoglires.

### Site-specific selection constraints and conserved domains

Our amino acid alignment of the 53 functional AMBN sequences revealed that many residues have been unchanged throughout mammalian evolution, some of them being regrouped into short domains (Additional file 2). Other conserved positions include amino acids that can be replaced with residues possessing the same characteristics (conservative positions). Unchanged and conservative positions are subjected to strong selective pressure, which indicates that they are functionally important for the protein. Most of them are located in the N-terminal region of the protein (encoded from exon 2 to beginning of exon 6). Conversely, more variable positions are located in the region encoded by exon 6, exon 7, and for a large part in exon 13 (Additional file 2).

**Fig. 5:**
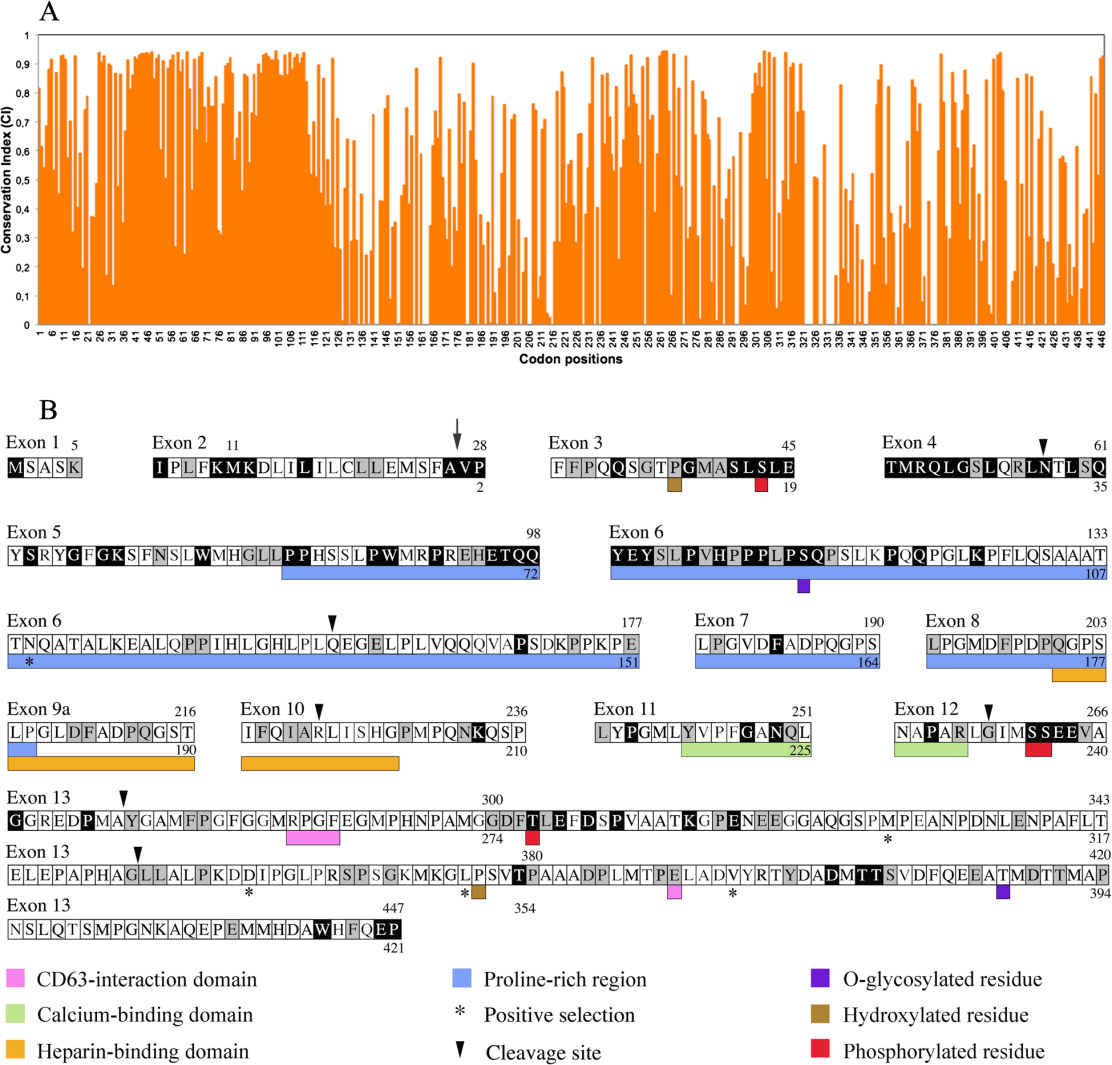
**a**. Estimation of the codon conservation index along the mammalian AMBN sequences. Positions 1 to 447 refer to the human sequence. Low evolutionary rates i.e., values close to 1, indicate the conserved / important positions, while high evolutionary rates i.e., values close to 0, indicate more variable positions. **b** Amino acid sequence of human AMBN. Human AMBN is composed of 447 residues when including the 26 residues of the putative leader peptide encoded by exons 1 and 2 (numbers above the residues); otherwise, 421 amino acids constitute the protein (numbers below the residues). Conserved positions are indicated either on black background (unchanged) or on gray background (only replaced with a residue having the same chemical property); variable positions are on white background. Various remarkable domains and remarkable residues are figured in color. The arrow points to the cleavage site of the signal peptide. Arrowheads point to proteolytic sites identified in porcine AMBN. The five sites under significant positive selection are indicated with an asterisk

Selective forces acting along the 53 *AMBN* codon sequences were inferred through non-synonymous vs. synonymous substitution rate (Fig. 5a). The comparison between the nearly neutral model (M7) and the model allowing a fraction of positively selected sites (M8) revealed significant among-site variation in dN/dS with the occurrence of positive selection (*p* < 0.001; Table 1). Under the M8 model, five codons out of 447 were identified as evolving under significant positive selection (PP > 0.95) during mammalian evolution. These sites are located in the proline rich region encoded by exon 6 at position 135, and in the exon 13 encoded region at positions 328, 360, 375, and 394 (Table 1, Fig. 5b).

The site conservation index calculated from the site-specific dN/dS values obtained under model M8 illustrated the variation of the selection constraints across the AMBN (Fig. 5a). Most of the N-ter region (positions 1 to 123, encoded from exon 1 to the 5' region of exon 6), several small regions dispersed in the AMBN sequence (notably positions 233–265, exons 10–12; 300–320, beginning of exon 13; 379–404, mid exon 13) and a short C-ter sequence (442–447, encoded by exon 13) are subjected to high functional constraints. Most of the remaining sequence is characterized by an alternation of high and low selective pressures (Fig. 5a). We identified 161 positions (out of 447 residues in human AMBN) subjected to purifying selection during more than 200 Ma of mammalian evolution pointing to biologically significant residues (Fig. 5b). These constrained positions have certainly important functions, which explains they were either unchanged (80 positions) or they displayed only few substitutions (81 positions). Among these residues are: the cleavage sites N^31^, G^232^, Y^249^ and G^326^/L^327^; the hydroxylated P^11^; the O-glycosylated S^86^; and the phosphorylated S^17^, S^235^, S^236^ and T^277^. In contrast, our study identified as variable positions, i.e., having probably no important function, the putative cleavage sites Q^130^ and R^196^, hydroxylated P^350^, and O-glycosylated T^387^ (Fig. 5b). Out of this dozen of important positions, there are 150 other selectively constrained positions that our evolutionary analysis pointed out as being important for the correct functioning of AMBN. Therefore, we predict that these conserved positions in AMBN, and particularly the unchanged ones, would be potentially responsible for enamel disorder if they were substituted.

Three conserved domains (or motifs) were previously identified as possibly playing an important functional or structural role (Fig. 5b). Between the two CD63-interaction domains the position E^364^ is conserved, while the RPGF motif is not. Half of the residues of the calcium-binding domain (Y^217^-R^230^) and eight residues out of 28 in the heparin-binding domain are conserved, which confirms that these domains are important. In addition to these domains, several conserved residues are located close one to the other in a same region of the protein, representing new motifs of unknown but important function. They are housed principally in the N-terminal region (residues 1–124) of the protein encoded by exons 3, 4, 5 and beginning of exon 6 (Fig. 5b). This large, conserved AMBN region contains important residues: the two first residues that are involved in the cleavage of the signal peptide, the hydroxylated P^11^, the phosphorylated S^17^, the O-glycosylated S^86^ and the cleavage site N^31^. The residues on both sides of these positions are also conserved, but in the same region other highly conserved amino acids constitute motifs of unknown function. We identified notably two highly conserved peptide sequences: the one is encoded by exons 3 and 4 (35 residues, 29 of them being conserved), and the other encoded by the 3' end of exon 5 and 5' extremity of exon 6 (22 residues conserved), that certainly play an important role remaining to be defined. Interestingly, it is known that the 5' end of exon 6 is alternatively spliced in the few human, rodent and porcine *AMBN* transcripts available. Our alignment revealed that the residue at the splicing site (Q^87^ = always encoded by CAG, not shown) is well conserved (Additional file 2). Similarly, several, highly conserved motifs encoded by exons 5 and 13 are highlighted as being probably important for the functionality of AMBN.

In mammals, AMBN does not possess a conserved RGD (integrin-binding) motif; however, we found an RGD sequence in the region encoded by the beginning of exon 13 in the cow and in four primates. This sequence was clearly acquired secondarily during mammalian evolution through the substitution RGG to RGD. The VTKG sequence (heparin-binding motif) is only present in rat, mouse, hamster and kangaroo rat, and was clearly acquired secondarily during rodent evolution through the substitution ATKG to VTKG. However, the TKG sequence is conserved in all eutherians and the lysine, K^288^, is unchanged (Fig. 5b). The DGEA motif previously described in rats and mice as a possible interacting site with integrins is also present in the hamster AMBN but not in other rodents. This motif was acquired secondarily during murid evolution.

### Lineage-specific selection constraints

**Fig. 6:**
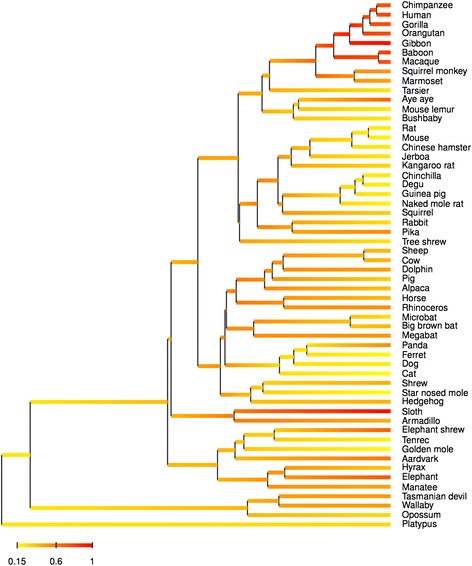
Bayesian reconstruction of dN/dS ratio in AMBN along the mammalian phylogeny. The variation of dN/dS was jointly reconstructed with divergence times while controlling the effect of three life-history traits (body mass, longevity, and maturity). The Bayesian chronogram revealed strong positive selection in catarrihine primates where elevated dN/dS ratios are inferred

The estimation of the global dN/dS ratio across the whole tree using the one-ratio branch model (M0) resulted in a ω value of 0.46 showing that AMBN is globally evolving under purifying selection in mammals (Table 1). The joint Bayesian reconstruction of the dN/dS ratio along the phylogeny revealed large variations among mammalian lineages with mean ω values of functional sequences ranging from 0.15 in cat to 1.03 in gibbon (Fig. 6). As expected, elevated ω values were found in the non-functional sequences of aardvark (0.65) and sloth (1.04) as a consequence of relaxed selection constraints, whereas the armadillo has a lower value (0.44). Conversely, some lineages with functional AMBN showed high dN/dS values such as elephant (0.74), elephant shrew (0.73), and aye-aye (0.71). However, AMBN has also evolved under strong purifying selection for instance in rodents with low values of ω observed in this group overall (0.19-0.32).

The most striking pattern in this reconstruction is nonetheless the high dN/dS ratios inferred along the whole catarrhine subtree, suggesting the occurrence of positive selection on AMBN in this particular group of Primates (Fig. 6). Hierarchical likelihood ratio tests performed between different branch models allowing ω to vary among branches of the phylogeny revealed that strong positive selection has occurred in Catarrhini (Table 1). Indeed, branch model LRTs for positive selection in the catarrhine subtree were strongly significant (*p* < 0.001) with both the 53-taxa and 56-taxa data sets, in which the dN/dS ratio in Catarrhini was estimated to be respectively 1.32 and 1.11. Finally, among the candidate branches revealed by the previous Bayesian analysis, two were estimated to have ω values higher than 1: in sloth (ω = 1.10), in which *AMBN* is pseudogenized, and in aye-aye (ω = 1.13), in which it nevertheless appears fully functional.

## Discussion

### A second translation initiation site (TIS) was recruited in an ancestral eutherian

Two TIS were clearly identified in our analysis as being conserved through mammalian evolution: one in exon 1, the other in exon 2. The latter is the ancestral TIS in tetrapod *AMBN;* it is the only TIS present in reptiles [14] and frogs [15]. This TIS is homologous to that of other SCPPs [1]. Interestingly, our analysis indicated also that the former TIS was recruited in the last common eutherian ancestor, then conserved in most lineages during more than 100 Ma of evolution [76]. This finding suggests that this additional TIS plays a role in enamel formation in most eutherian species, and contradicts previous studies proposing that the only functional TIS was that in exon 2 [9, 32]. The presence of two (predicted) functional TIS means, however, that they do function through alternative splicing of exon 1 and that this process could play a role in the regulation of the amount of protein synthesized from the mRNA [66, 67]. Such alternative splicing may result in the presence of two AMBN isoforms: one with a short, 16 amino acid long, signal peptide (SP) and the other with a long SP (26 residues), the function of which remains to be tested. The presence of two signal peptides (SP) is quite common in proteins, in which two SP could play a role in the export efficiency of the protein [93]. During amelogenesis, the either use of these SP could regulate AMBN secretion in the forming enamel matrix. For further discussion on this topic we refer the readers to our previous study on ENAM, in which two functional TIS were similarly identified in two exons (exons 3 and 4), resulting in a short and a long SP [54]. The second, short SP of AMBN is homologous to the second SP described in ENAM [1], and sequence similarity in the N-terminal region of these two EMPs allowed to propose that *AMBN* was derived from an ancestral *ENAM* after duplication [4]. In fact, *AMBN* exon 2 and *ENAM* exon 4 are homologous exons, and both possess 15 nucleotides upstream the TIS. In contrast, *AMBN* exon 1 and *ENAM* exon 3, in which is located the first TIS, are not homologous. They were recruited separately during evolution. In *ENAM* the first TIS was recruited in a mammalian ancestor more than 200 Ma [94], while in *AMBN* we showed here that it was recruited later, in an eutherian ancestor, more than 100 Ma. In mammals, the convergent recruitment of an additional TIS upstream the ancestral one in two related enamel matrix proteins is, however, intriguing. Indeed, one can wonder whether the second event i.e., the recruitment of a large SP in AMBN, was linked to the presence of two SP in ENAM, and what was the benefit of this feature for enamel microstructure. To our knowledge there are no data in the literature showing to what extend the relative amount of the two EMPs, AMBN and ENAM, is important for enamel microstructure, nor whether the two proteins interact. Here, we speculate that the TIS responsible for encoding a larger SP has appeared at random in *AMBN* exon 1, then was conserved as providing a better fit with the different regulation of ENAM secretion through the presence of its two SP.

### A hotspot for DNA duplication has appeared in placental AMBN

As previously reported in the human *AMBN*, we identified additional exons of same length and similar sequence in the same region of 12 unrelated eutherian *AMBNs*. We concluded that these additional exons resulted from tandem duplications of the region containing exon 7 and, therefore, are all homologous. Because the duplication of this short DNA region occurred repeatedly and independently in various lineages, we consider that the *AMBN* region located downstream exon 7 is a hotspot for the occurrence of such duplication. The process of tandem duplication is known as a major mechanism for gene elongation, but is it advantageous for AMBN function or simply neutral? Repeated duplication of exon 7 was found in terminal taxa, except in simiiform primates, in which the first duplication of this region could have occurred in their common ancestor. The duplicated region was conserved (and some copies were added) in all simiiform gDNA studied excepted in the gorilla genome, in which either the duplicated region was secondarily lost or this *AMBN* region was badly assembled. Given the presence of these duplicated exons in various taxa and their conservation in simiiform lineages one can wonder whether the region they encode could not have some selective advantage. In addition to enlarge the protein with 13 residues, each exon encodes two to four prolines. Because these duplications do not change much the high percentage of proline in this region, this is the increase in length of this proline-rich region that could be advantageous. Indeed, it has been suggested that the length of the proline-rich region of the EMPs could determine the supramolecular enamel matrix assembly [95].

### Alternative polyadenylation signals (PS) were conserved through mammalian evolution

The 3'UTR of a mRNA is important and anomalies in this region of the mRNAs are implicated in a variety of human diseases [96]. Here, we identified two to five PS in the 3' UTR of the sequences analyzed. Three of them were present in the ancestral mammalian *AMBN* and conserved in all sequences during mammalian evolution. These variant PS could also be selected for regulatory purposes in the concerned sequences. For instance, it is known that mRNAs with multiple PS tend to use noncanonical signals (i.e., ATTAAA) more often than mRNAs with a single PS [97]. The long lasting conservation of the three PS through 200 Ma means that *AMBN* codes for three mRNAs that differ in their 3' end and play an important role in post-transcriptional regulation. This finding partially confirms previous reports of two *AMBN* transcripts in rodents and humans: a short sequence ending after PS1 and a large sequence ending after PS5 [9, 11, 13]. In rodents, one of these polyadenylation sites shortens the sequence at the 3' end, which could explain the presence of two transcripts. Here we show that a third transcript sequence ending at PS3 could also exist. The presence of multiple PS is significantly observed in mammalian mRNAs, and alternative polyadenylation may also influence the stability, translation efficiency, or localization of an mRNA [98, 99]. Indeed, mRNAs vary greatly in stability depending on the length of the 3'UTR, which changes a.o., the number of potential binding sites for microRNAs. However, to our knowledge *AMBN* mRNAs were not found among targets of several miRNAs [100].

### Selection analyses highlighted residues and domains important for AMBN function

Our evolutionary analysis revealed that most cleavage sites and most hydroxylated, phosphorylated and O-glycosylated residues either identified or predicted in previous studies are included into the 161 conservative positions that were found here subjected to purifying selection (i.e., functionally constrained) during 200 Ma of mammalian evolution. They are for instance, the hydroxylated P^11^, phosphorylated S^17^ (SxE motif) and S^86^, and the cleavage site N^31^. These findings, therefore, confirm that these amino acids are important for the AMBN function and, vice versa, given that these positions were previously identified as probably important they support evolutionary analysis as an efficient method for detecting important residues. Similar studies targeting AMEL or ENAM led to the same findings [53, 54]. Interestingly, most of these remarkable residues are adjacent to unchanged amino acids, which indicate that the latter certainly play a role in stabilizing the environment of the crucial residue. Also, our analysis raised some doubts about the potential function of a few positions that were previously predicted as being important in studies of rat and pig AMBN, i.e., some cleavage sites [5, 6, 101, 102], and other hydroxylated and O-glycosylated residues [29, 103–105]. However, conservation of specific sequences could not be necessary for the cleavages to occur.

The proline-rich and calcium-binding domains do not display a large number of unchanged positions but each possesses some conservative positions that probably play a role in the function of the domain. It is known that such domains do not need high sequence conservation but rather a general context appropriate for their function. Proline-rich sequences are known to bind WW and SH3 domains, two small protein modules that mediate protein-protein interactions and are key parts of signaling cascades [106–108]. The calcium-binding site (i.e., an helix-loop-helix structural domain known as EF-hand motif) is supposed either to be involved in mineral nucleation or in calcium-mediated cell binding [23, 29, 30]. Some motifs reported in rodent AMBN, such as VTKG [7, 49, 109] and DGEA [110] are not conserved in mammalian evolution. Given that these motifs are known to interact with cell surface proteins (integrins) they are supposed to serve to link the ameloblasts to the forming enamel matrix. However, their absence in most mammalian lineages suggests that these motifs were only acquired in the rodent lineage ancestor and could have a functional role solely in rodents.

Remarkably, conserved positions and the adjacent conserved residues are however few compared to the 150 positions that were highlighted in our evolutionary analysis, the function of which remain to be characterized. Two important motifs were identified by their high number of conserved residues in the region encoded by exons 3 and 4, on the one hand, and by the 3' end of exon 5 and the 5' extremity of exon 6, on the other hand. Both motifs contain important residues; the first one houses hydroxylated P^11^, phosphorylated S^17^ (SxE motif) and cleavage site N^31^. It remains to be determined whether the other conserved positions are only important in order to keep these residues functional or have other functions. The second motif contains a remarkable residue, a O-glycosylated S^86^. Here also one can wonder what could be the function of the other conserved positions. In addition, previous studies have revealed that the 15 amino acid sequence encoded by the 5' extremity of exon 6 (including the O-glycosylated S^86^) is subjected to alternative splicing in rodents, human and pig *AMBN* through the presence of an intraexonic splicing site [6, 11, 12]. Here we showed that the residue at the splicing site (Q^87^ = CAG) is conserved in mammalian evolution, which strongly suggests that the two isoforms resulting from the splicing play distinct but crucial roles for the correct functioning of AMBN. In particular, it is worth noting that (i) the short isoform does not contain this O-glycosylated position, suggesting avoiding interaction with the cell membrane is important to be able to fulfill its function, and (ii) deletion of exon 6 is associated with amelogenesis imperfecta [48] with hypoplastic enamel as previously reported in mice lacking *AMBN* exons 5 and 6 (see below) [111].

### Identification of disease-associated mutations potentially leading to AI

A total of 161 positions were identified in mammalian AMBN as conservative positions, i.e., residues that were unchanged or only substituted with a residue having the same properties during more than 200 Ma of mammalian evolution. This finding leads to the prediction that the amino acids located at these positions play an important role. They cannot be changed otherwise the AMBN function linked to this position will be disturbed and will result in a genetic disease with apparent enamel disorder. In fact, the process of natural selection provides us with tests in nature, and our previous evolutionary analyses performed on various SCPPs, including AMELX, ENAM, MEPE, AMTN, and DMP1, have shown that the patterns of long-term evolutionary conservation are crucial for validating human genetic diseases related to residue substitutions [53–57]. Such patterns were recently used for *in silico* functional diagnoses of non-synonymous single nucleotide variants found in thousands of disease-associated genes (see review in [58]). Given these various findings, we predict that *AMBN* is an excellent candidate for enamel genetic disease, amelogenesis imperfecta (AI). This prediction confirmed the conclusions previously reached by many authors because (i) *AMBN* was located on chromosome 4 in a region containing the locus for AIH2, the autosomal hypoplastic form of AI [12, 47], and (ii) the important function of AMBN during amelogenesis, as illustrated by *AMBN*^−/−^ mice that exhibit severe enamel hypoplasia [49].

During years, mutations of *AMBN* were never found associated with AI phenotypes until recently when hypoplastic AI was associated with homozygous exon 6 deletion [48]. The reason why other AMBN-associated AI were not identified could be due to an autosomal recessive pattern of inheritance, and this is supported by the fact that *AMBN*^+/−^ mice do not display any dental phenotype [49]. Another reason could be that AMELX compensate, at least partially, for AMBN deficiency. The two encoding genes are phylogenetically related, they share some similarities, notably in the 5' region, and both proteins possess a proline-rich region [4, 112]. Therefore, in case of homozygous mutation of AMBN compensation by AMELX could contribute to weaken the dental phenotype, which could be hardly detectable.

Deletion of exon 6 leads to the lack of 14 highly conserved and 11 conservative residues, among which the O-glycosylated S^86^, and a large part of the proline-rich region of the protein. Given this number of sensitive positions predicted by our evolutionary analysis, the mutation is validated as being responsible for the AI and this region as playing an important role during enamel matrix formation.

### Lineage-specific positive selection and relaxation of functional constraints

Analyses of lineage-specific selective constraints acting on functional AMBN sequences have revealed a large variation in dN/dS ratio across mammals, even though this gene globally evolved under purifying selection. One of our most prominent results is the strong signal for positive selection detected in catarrhine primates, in which the dN/dS ratio was estimated to be greater than 1. This suggests that episodes of positive selection have occurred throughout the evolutionary history of this sub-clade. Interestingly, this elevated dN/dS ratio correlates with the presence of additional *AMBN* exons in several of these primate species suggesting that these exon duplications might be adaptive. Apart from Catarrhines, we also found evidence for positive selection in AMBN along the aye-aye (*Daubentonia madagascariensis*) lineage.

In Primates a correlation between enamel thickness and diet has been reported [113] and enamel thickness is often used to infer the diet of both extant and fossil primates [114, 115]. Adaptive changes related to diet shifts in Primates have been reported in another enamel protein (ENAM) and it has been hypothesized that differences in tooth enamel thickness were correlated with the adaptive evolution of enamelin [116]. Moreover, the aye-aye differs from other lemurs in possessing rodent-like gnawing and ever-growing incisors, and molars with a particularly thick enamel layer [115]. Our results therefore suggest that adaptive changes might have occurred in AMBN of catarrhines and aye-aye in response to selective constraints imposed by dietary adaptation through changes in enamel thickness.

Our analyses also detected traces of relaxed selection in enamel-less species: aardvark, armadillo, and sloth. In adult aardvarks (Afrotheria), armadillos and sloths (Xenarthra), teeth indeed lack enamel. In the nine-banded armadillo (*Dasypus novemcinctus*), however, at birth the teeth are covered with a thin layer of enamel that is no longer present in adults [61, 117]. In birds and in various tooth- or enamel-less mammals, selective pressures on enamel-specific proteins were relaxed after the ability to form enamel was lost, and the genes were inactivated, becoming pseudogenes [39–41, 118, 119]. As expected from these previous studies, we showed that *AMBN* has accumulated random deleterious mutations resulting in stop codon and frameshifts in sloth and aardvark, which confirms that AMBN is an enamel-specific protein. In the nine-banded armadillo, the *AMBN* sequence has not drastically changed, a condition that could be expected when considering the presence of a thin enamel cover in the young, which strongly suggests that AMBN could be expressed during enamel formation prior to birth. However, we identified several substitutions at AMBN positions that were considered important for the function of the protein. Such mutations could lead to the expression of a defective protein resulting in the deposition of a thin (hypoplastic?) enamel layer at the tooth surface, which subsequently disappears after birth through abrasion. Further structural and molecular studies of enamel layer formation in this armadillo species are however needed in order to confirm or infirm this hypothesis.

## Conclusions

By adding 50 new sequences to the six previously published sequences, our study improved considerably our knowledge on the gene structure, protein composition and evolution of AMBN in mammals. In particular, we revealed that (i) an additional TIS was recruited in an ancestral eutherian *AMBN*, allowing the translation of a second, alternative, large SP, (ii) a short DNA sequence including exon 7 was duplicated several times in various eutherian species, increasing the proline-rich region, (iii) several PS were functional, suggesting a regulation process in the 3'UTR, (iv) numerous residues were conserved during more than 200 Ma of mammalian evolution, which strongly suggests that they are structurally and/or functionally important for the correct function of the protein, (v) several conserved residues constituted new domains of predicted high importance, and (vi) *AMBN* was invalidated in enamel-less species as previously reported [40, 41]. The putative function of some residues identified in previous studies was not confirmed by our analyses, and cannot be generalized to all mammals. Finally, the presence of highly conserved residues indicates that *AMBN* is a good candidate gene for amelogenesis imperfecta.

## Additional files

Additional file 1:
**Names of the 56 mammalian species used in this study.** Preferred common names, scientific names, families, orders and references in GenBank are listed in alphabetical order of common names. The *AMBN* sequence of the crocodilian *Caiman crocodilus* was used as outgroup. (PDF 95 kb) Additional file 2:
**Amino acid alignment of the 53 mammalian AMBN used in our evolutionary analysis.** The sequences were aligned against the human sequence and are ordered following mammalian relationships. Our alignment led to 500 positions, including gaps. For convenience of presentation our alignment does not include the duplicated exons 9c, 9d and 9e (+9c; +9c-9e) that were found in three species only. The specific length of each AMBN in indicated in brackets at the end of the sequence. The signal peptide (underlined) can start at the methionine located either in the region encoded by exon 1 or exon 2 (M, squared). For a better understanding of SP evolution, the sequences in which the first methionine is lacking are highlighted on grey background. Exon limits are indicated by vertical lines; (.): residue identical to human AMBN residue; (−): indel; (?): unknown amino acid; *: stop codon. (PDF 138 kb) Additional file 3:
**Comparison of intron/exon boundaries and UTR of twelve mammalian** ***AMBN*** **sequences.** For scientific names and references, see Additional file 1. (//): sequence not shown; (?): unknown nucleotide; (.): nucleotide identical to human *AMBN* nucleotide; (−): indel. (PDF 143 kb) Additional file 4:
**Prediction of functional signal peptides (SP) in the AMBN sequence using** ***Signal P*** **software.** Here the human sequence was used as an example. The high probability for the two sequences (score = 0.998) indicates that the SP encoded either by exon 1 + exon 2 (26 residues) (**A**) or by exon 2 only (16 residues) (**B**) are functional. The cleavage site (arrow) does not vary in the two sequences analyzed and exhibits a similar, high probability: 0.971 in **A**, between positions 26 and 27; 0.965 in **B**, between positions 16 and 17. The three characteristic regions of SP (n-, h- and c-regions) are indicated. (TIFF 488 kb) Additional file 5:
**Amino acid composition of AMBN.** The amino acid percentages are given for the full length sequence and the proline-rich region for six representative mammalian AMBN, the putative ancestral mammalian sequence, and the crocodile, and are compared to average frequency in most proteins. The percentages of proline and glutamine are in bold characters. (PDF 100 kb)
