# Supplementary material for: Evolutionary analysis of selective constraints identifies ameloblastin (AMBN) as a potential candidate for amelogenesis imperfecta
Source: BMC Evol Biol. 2015 Jul 30;15:148. doi: 10.1186/s12862-015-0431-0 (PMC4518657; doi:10.1186/s12862-015-0431-0)
Supplement: Additional file 1: — Names of the 56 mammalian species used in this study. Preferred common names, scientific names, families, orders and references in GenBank are listed in alphabetical order of common names. The AMBN sequence of the crocodilian Caiman crocodilus was used as outgroup. (PDF 95 kb) [file 12862_2015_431_MOESM1_ESM.pdf]

Additional file 1. Names of the 56 mammalian species used in this study.
Preferred common names, scientific names, families, orders and references in GenBank for the 56 mammalian species, from which *AMBN* sequences were used in our study. Alphabetical order of common names. The AMBN sequence of the crocodilian *Caiman crocodilus* was used as an outgroup.

Common name	Genus and species	Family	Order	Source
Aardvark	*Orycteropus afer*	Orycteropodidae	Tubulidentata	XM_007942818.1
Alpaca	*Vicugna pacos*	Camelidae	Cetartiodactyla	ENSVPAG00000011879
Armadillo	*Dasypus novemcinctus*	Dasypodidae	Cingulata	ENSDNOG00000010874
Aye-Aye	*Daubentonia madagascariensis*	Daubentoniidae	Primates	DauMad_1.0
Baboon	*Papio anubis*	Cercopithecidae	Primates	ENSPANG00000014745
Big brown bat	*Eptesicus fuscus*	Vespertilionidae	Chiroptera	XM_008157477.1
Bushbaby	*Otolemur garnettii*	Galagidae	Primates	ENSOGAG00000033618
Cat	*Felis catus*	Felidae	Carnivora	ENSFCAG00000009206
Chimpanzee	*Pan troglodytes*	Hominidae	Primates	ENSPTRG00000023351
Chinchilla	*Chinchilla lanigera*	Chinchillidae	Rodentia	XM_005392786.1
Chinese hamster	*Cricetulus griseus*	Cricetidae	Rodentia	XM_007624123.1
Cow	*Bos taurus*	Bovidae	Cetartiodactyla	NM_173988
Degu	*Octodon degus*	Octodontidae	Rodentia	XM_004646538.1
Dog	*Canis familiaris*	Canidae	Carnivora	ENSCAFG00000002904
Dolphin	*Tursiops truncatus*	Delphinidae	Cetartiodactyla	ENSTTRG00000008661
Elephant	*Loxodonta africana*	Elephantidae	Proboscidea	ENSLAFG00000016997
Elephant shrew	*Elephantulus edwardii*	Macroscelididae	Macroscelidea	XM_006896844.1
Ferret	*Mustela putorius furio*	Mustelidae	Carnivora	ENSMPUG00000009583
Golden mole	*Chrysochloris asiatica*	Chrysochloridae	Afrosoricida	XM_006873792.1
Gibbon	*Nomascus leucogenys*	Hylobatidae	Primates	ENSNLEG00000007704
Gorilla	*Gorilla gorilla*	Hominidae	Primates	ENSGGOG00000013657
Guinea pig	*Cavia porcellus*	Caviidae	Rodentia	AJ537436
Hedgehog	*Erinaceus europaeus*	Erinaceidae	Erinaceomorpha	ENSEEUG00000011188
Horse	*Equus caballus*	Equidae	Perissodactyla	ENSECAG00000021532
Human	*Homo sapiens*	Hominidae	Primates	NM_016519
Hyrax	*Procavia capensis*	Procaviidae	Hyracoidea	ENSPCAG00000003170
Jerboa	*Jaculus jaculus*	Dipodidae	Rodentia	XM_004665362.1
Kangaroo rat	*Dipodomys ordii*	Heteromyidae	Rodentia	ENSDORG00000016295
Macaque	*Macaca mulatta*	Cercopithecidae	Primates	ENSMMUG00000022099
Manatee	*Trichechus manatus*	Trichechidae	Sirenia	XM_004383356.1
Marmoset	*Callithrix jacchus*	Cebidae	Primates	ENSCJAG00000014634
Megabat	*Pteropus vampyrus*	Pteropodidae	Chiroptera	ENSPVAG00000004326
Microbat	*Myotis lucifugus*	Vespertilionidae	Chiroptera	ENSMLUG00000012042
Mouse lemur	*Microcebus murinus*	Cheirogaleidae	Primates	ENSMICG00000005341
Mouse	*Mus musculus*	Muridae	Rodentia	NM_009664
Naked mole rat	*Heterocephalus glaber*	Bathyergidae	Rodentia	XM_004871725.1
Opossum	*Monodelphis domestica*	Didelphidae	Didelphimorphia	ENSMODG00000012395
Orangutan	*Pongo pygmaeus*	Hominidae	Primates	ENSPPYG00000014807
Panda	*Ailuropoda melanoleuca*	Ursidae	Carnivora	ENSAMEG00000005828
Pig	*Sus scrofa*	Suidae	Cetartiodactyla	NM_214037
Pika	*Ochotona princeps*	Ochotonidae	Lagomorpha	ENSOPRG00000010951
Platypus	*Ornithorhynchus anatinus*	Ornithorhynchidae	Monotremata	ENSOANG00000013691
Rabbit	*Oryctolagus cuniculus*	Leporidae	Lagomorpha	ENSOCUG00000022916
Rat	*Rattus norvegicus*	Muridae	Rodentia	NM_012900
Rhinoceros	*Ceratotherium simum*	Rhinocerotidae	Perissodactyla	XM_004419143.1
Sheep	*Ovis aries*	Bovidae	Cetartiodactyla	ENSOARG00000011393
Shrew	*Sorex araneus*	Soricidae	Soricomorpha	ENSSARG00000009204
Sloth	*Choloepus hoffmanni*	Megalonychidae	Pilosa	ENSCHOG00000011594
Squirrel	*Ictidomys tridecemlineatus*	Sciuridae	Rodentia	ENSSTOG00000007952
Squirrel monkey	*Saimiri boliviensis*	Cebidae	Primates	XM_003931927.1
Star-nosed mole	*Condylura cristata*	Talpidae	Soricomorpha	XM_004681235.1
Tarsier	*Tarsius syrichta*	Tarsiidae	Primates	ENSTSYG00000014624
Tasmanian devil	*Sarcophilus harrisii*	Dasyuridae	Dasyuromorphia	ENSSHAG00000018085
Tenrec	*Echinops telfairi*	Tenrecidae	Afrosoricida	ENSETEG00000002140
Tree shrew	*Tupaia chinensis*	Tupaiidae	Scandentia	ENSTBEG00000017478
Wallaby	*Macropus eugenii*	Macropodidae	Diprotodontia	ENSMEUG00000002847
Crocodile	*Caiman crocodilus*	Crocodylidae	Archosauria	AY043290
