# Supplementary material for: Evolutionary analysis of selective constraints identifies ameloblastin (AMBN) as a potential candidate for amelogenesis imperfecta
Source: BMC Evol Biol. 2015 Jul 30;15:148. doi: 10.1186/s12862-015-0431-0 (PMC4518657; doi:10.1186/s12862-015-0431-0)
Supplement: Additional file 2: — Amino acid alignment of the 53 mammalian AMBN used in our evolutionary analysis. The sequences were aligned against the human sequence and are ordered following mammalian relationships. Our alignment led to 500 positions, including gaps. For convenience of presentation our alignment does not include the duplicated exons 9c, 9d and 9e (+9c; +9c-9e) that were found in three species only. The specific length of each AMBN in indicated in brackets at the end of the sequence. The signal peptide (underlined) can start at the methionine located either in the region encoded by exon 1 or exon 2 (M, squared). For a better understanding of SP evolution, the sequences in which the first methionine is lacking are highlighted on grey background. Exon limits are indicated by vertical lines; (.): residue identical to human AMBN residue; (−): indel; (?): unknown amino acid; *: stop codon. (PDF 138 kb) [file 12862_2015_431_MOESM2_ESM.pdf]

Additional file 2. Amino acid alignment of the 53 mammalian AMBN used in our evolutionary analysis.

The sequences were aligned against the human sequence and are ordered following mammalian relationships [117]. Our alignment led to 500 positions, including gaps. For convenience of presentation our alignment does not include the duplicated exons 9c, 9d and 9e (+9c; +9c-9e) that were found in three species only. The specific length of each AMBN in indicated in brackets at the end of the sequence. The signal peptide (underlined) can start at the methionine located either in the region encoded by exon 1 or exon 2 (M, squarred). For a better understanding of SP evolution, the sequences in which the first methionine is lacking are highlighted on grey background. The 15 alternatively spliced residues encoded by the 5' end of exon 6 in rodents, human and pig are indicated in bold. Exon limits are indicated by vertical lines; (.): residue identical to human AMBN residue; (-): indel; (?): unknown amino acid; *: stop codon.

```
                   ex 1         exon 2        1            exon 3                exon 4                         exon 5                        exon 6      122
Human              MSASK | IPLFKMKDLILILCLLEMSFAVP--- | FFPQQSGT-----PGMASLSLE | TMRQLGSLQRLNTLSQ | YSRYG-FGKSFNSLWMHGLLPPHSSLPWMRPREHETQQ | YEYSLPVHPPPLPSQ
Chimpanzee         ..... | .......................--- | ........-----......... | ................ | .....-................................ | ...............
Gorilla            ..... | .......................--- | ........-----......... | ................ | .....-................................ | ...............
Orangutan          ..... | .......................--- | ........-----......... | ................ | .....-................................ | ...............
Gibbon             ..... | .......................--- | ...R....-----......... | .........G...... | .....-.........L...................... | ...............
Baboon             ..... | .......................--- | .....T..-----......... | .........G...... | ...F.-...................P............ | ...............
Macaque            ..... | .......................--- | .....T..-----......... | .........G...... | ...F.-....L..............P............ | ...............
Squirrel monkey    ..P.. | .............G..K......--- | L.......-----......... | .........G...... | ...F.-...................F............ | ...............
Marmoset           ..... | .............G.........--- | L.......-----......... | .........G...... | ...F.-...................F............ | ...............
Aye-aye            ..... | ...................V...--- | V..K...I-----......... | ......T..G..A... | F..F.-..........ND.......F............ | ...............
Bushbaby           TA... | .S.....G.L.T.WF.HV.A...--- | V......V-----......... | .........G...... | ...F.-.........LQ.....QP.............. | ...............
Mouse lemur        ..E.. | ..............F.H..V...--- | V......I-----......... | .........G...... | F..F.-..........N....Q...F............ | ...............
Tarsier            ..... | ................D.G....--- | V....P..-----......... | .........G...... | ...F.-...................F............ | ...A..........L
Tree shrew         ..... | ...C..............C....--- | M....P..-----......... | .........G...... | .....-..........Q........F............ | ...............
Kangaroo rat       ..... | ...........F....K......--- | A....P..QGMAP......... | .........G..A... | ...F.-...AL....L......PA.F.....G...... | ??????????.....
Rat                ..... | .......G.L.F.S.VK..L...--- | A....P.AQGMAP......... | .........G..A... | ...L.-...AL....L.......N.F..IG........ | ...............
Mouse              ..... | .......G...F.S.VK..L...--- | A....P.AQGMAP......... | .........G..A... | ...L.-...AL....L.......N.F..IG........ | ...............
Chinese hamster    ..... | .......G...F.S..K..L...--- | A....P.AQGMAP......... | .........G..A... | ...L.-...AL.T..L.......N.F..I......... | ...............
Jerboa             .P.A. | .......G.V.F....K......--- | A....P.AQNMAP......... | .........G..A... | ...L.-...A..T..LQ.....P..F..I..T...... | ...............
Naked mole rat     ..P.. | ...........L....K.T....--- | A....P.A-NMAP......... | .........G..A... | ...L.-....L....LQ.....P..F..L......... | ....V..........
Chinchilla         ..P.. | .........M.L.S..K......--- | A....P.A-NMAP......... | .........G..A... | ...L.-L...L....LQ.....P..F..L......... | ....V..........
Degu               ..P.. | .........M.L.F..K......--- | A....P.A-NMAP......... | .........G..A... | ...F.-....L....LQ.....P..F..L......... | ....V..........
Guinea pig         ..P.. | .........M.F.S..KI.....--- | A....P.A-NMAP......... | .........G..A... | ...F.-....L....LQ.....P..F..L......... | ....V..........
Squirrel           ..... | .SF....................--- | M....P.A-GMAP......... | .........G..A... | F..F.-....L....L............L..T...... | ...............
Rabbit             ..... | ...........L.....T.....--- | A....P..-----......... | .........G..A... | ..K..-....L....LQ.....P..F............ | ...............
Pika               ..E.. | .......G...L....GT.....--- | A....P..-----......... | .........G..A... | L.KF.Q....L....L......P..F.......Q.... | ......E.L......
Cow                .P.L. | ........M.......K..S...--- | A....P.I-----......... | .........G..L... | ...F.-..........N........F............ | ...............
Sheep              .P.L. | ........V.......K..S...--- | A....P.I-----......... | .........G..L... | ...F.-..........N..................... | ...............
Dolphin            .P.L. | ........M.......K..S...--- | V....P..-----......... | ........LG..M... | ...F.-...................F............ | ...............
Pig                .P.L. | ........MV......K..S...--- | A..R.P..-----..V...... | .........G..M... | ...F.-...................FQ........... | ...............
Alpaca             .P.L. | ........M.......K..S...--- | A....P..-----......... | .........G..L... | ...F.-...................F............ | ...............
Horse              .P.L. | .........V........GS...--- | A..R.P.I-----......... | .........G..M... | ...F.-Y.........N.....P..F............ | ...............
Rhinoceros         .P.L. | .........V.........S...--- | V....P..-----......... | .........G..M... | ...F.-.........L.........F............ | ...............
Dog                ...L. | .........V......K..S...--- | V....P..-----......... | .........G..M... | ...F.-...................F............ | ...............
Panda              ...L. | ...S............G..S...--- | V..R.P..-----......... | .........G..M... | ...F.-...................F............ | ...............
Ferret             ...L. | ..............V....S...--- | V....P..-----......... | .........G..M... | ...F.-...................F............ | ...............
Cat                ..TL. | ...S........F......S...--- | M....P..-----......... | .........G..M... | ...F.-...................F............ | ...............
Shrew              .P.LQ | ...I...............S...--- | V..K.A.I-----......... | .........G..M... | ..KF.-.........LQ........F.....K...... | ...............
Star-nosed mole    .P.L. | .S.......M.......L.S...--- | M....P..-----......... | .........G..M... | ...F.-...................F............ | ...............
Hedgehog           HASIE | D............Y....CS...--- | V..K.P.M-----......... | .........G..M... | ...F.-..........N.....NP.F............ | ...............
Megabat            GQH*R | SHFI...............Y...--- | V.......-----......... | ......T.HG..M... | ..KF.-...........S.......F............ | ...............
Big brown bat      GPE.T | .......N...V.....T.S...--- | V..R.P..-----A........ | ......T..G..M... | ...F.-....L.....Q........F........G... | ...............
Microbat           RP.L. | .......N...V....GT.S...--- | V....P..-----......... | ......T..G..M... | ...F.-....L..............F............ | ...............
Tenrec             ...L. | ???????????????????????--- | M..R.P..-----......... | .........G..I.P. | ...F.-...................F............ | ...............
Golden mole        ...L. | ............M...AVG....--- | M....P..-----......... | .........G..M.P. | ...F.-...................F............ | ...............
Elephant shrew     ...L. | ........P..FM...GV.V...--- | V....P..-----......... | ......R..G..I.P. | F..F.-......P..L.........F..L...D..... | ...............
Hyrax              ...L. | ?????.......M...GV.C...--- | V...---.-----......... | .........G...... | ...F.-.......M..........PF............ | ...............
Elephant           L..LQ | ............M...R......--- | V....P..-----......... | .........G...... | ...F.-....L....V........AF............ | ...............
Manatee            ...L. | .......N....S...GV.....--- | M....P..-----......... | .........G..M... | ...F.-.......M.L........PF............ | ...............
Opossum            KPVV. | KS.......L.M....GT.....IKQ | VY...AAA-----T.LG..... | ......N.NG..L... | F..F.-Y..P.T.............F...QQ....... | ...A...........
Tasmanian devil    KP.V. | KS.......L.M....GTC....IKQ | V....AAA-----T.LG..... | ......N.NG..L... | F..F.-...Q..P............F...QQ....... | ...A...........
Wallaby            KP.V. | ???????????????.GT.....IKQ | V....AAA-----T.LG..... | ......N.NG..L... | L..L.-Y..P.T.......V.....F..IQQ....... | ...A..L........
Platypus           QV.G. | G.TL..EN.V...S.FGT...I.--- | V.L.PG.P-----...G..... | ...H..N---..L.P. | F..F.-...Q........F.....NY..LS..DQ.... | ...A.......R...
```

	exon 6	exon 7	exon 8	exon 9a	exon 9b	245
Human	PSLKPQQPGLKPFLQSAAATT-NQATALKEALQPPIHLGH-LPLQEGEL-PLV---QQQVAPSDKPPK-PE	LPGVDFADPQGPS	LPGMDFPDPQGPS	LPGLDFADPQGST	-------------	
Chimpanzee	.....................-.................Q-........-...---............-..	....	....	....	-------------	
Gorilla	.....................-.................Q-........-...---............-..	...L...H...ST	-------------	-------------	-------------	
Orangutan	.....................-.................Q-........-...---..........L.-..	....	...L..A....ST	-------------	-------------	
Gibbon	............L.......A-.................Q-........-...---............-..	..R..........	....	...........P.	-------------	
Baboon	...............A....A-DE...Q...........Q-.L......-...---............-..	...A.V......P	..R...A......	......P....PS	LPRMDFADPQGPS	+9c
Macaque	...............A....A-DE...Q....R......Q-.L......-...---............-..	...A.V......P	..R..........	..RM..P....PS	LPGLDFPDPQGPS	+9c
Squirrel monkey	..................G.A-.....Q.-.........Q-.L......-.PI---E...........-..	...I.........	..R...A...D..	I..........PS	-------------	
Marmoset	.........M........G.A-...S.Q.-........EQ-........-.PI---E...........-..	...I.........	..R...A......	..R........PS	-------------	
Aye-aye	...Q...A......PPNG..A-V....Q.GRP....R.EQ-...K...P-.AI---E...........-..	..VM..P......	-------------	-------------	-------------	
Bushbaby	.A.Q...A.......PT...A-V.G..Q.GRP...V...Q-........-.A.---.E....EE.L..-..	..IM...G.....	-------------	-------------	-------------	
Mouse lemur	...Q...A.......PT..NA-I...EQ.GGP...A...Q-........-.AI---..PA........-..	..AM...H.....	-------------	-------------	-------------	
Tarsier	.NQ.....V..ALH.PT...A-L...EQQGEA....L..Q-.......V-.AIKQI............-L.	..VM.....R...	-------------	-------------	-------------	
Tree shrew	...Q...........PT...A-I....Q.GG.....R.EE-...KQA..-.AL---......A.....-..	..RLE..V.P..T	-------------	-------------	-------------	
Kangaroo rat	.P.H.....M.....PT.S.A-F...VQ.GGPH..M.P.Q-...KD..M-.A.---.......E...T-S.	M.LM...GQEL.T	-------------	-------------	-------------	
Rat	...Q.H.........PT...G-V.V.PQ.PGPH..M.P.Q-........IAPD---EP.....EN..T-..	V.IM......F.T	-------------	-------------	-------------	
Mouse	...Q.H.........PT...G-V.V.PQ.PGP...M.P.Q-........IAPD---EP.....EN..T-..	V.IM......F.T	-------------	-------------	-------------	
Chinese hamster	...Q.H.........PTD..G-V.V.PQ.PGP...M.P.Q-........TAADE--.P..L..EN..T-..	.V.LM......F.	-------------	-------------	-------------	
Jerboa	.....H.........PT...A-..D..Q.GGP...M.P.Q-........TADQ---..E....E...T-..	M.IM......F.S	-------------	-------------	-------------	
Naked mole rat	..QQ.P.......Q.PT.TPA-V.D.PP.GGP...M.PRQ-...KD...-.EA---H.....TEMALN-..	..VL......I..	-------------	-------------	-------------	
Chinchilla	.PQQ.P.......Q.PT.TPA-V.DAPP.GGP...M.PRQ-...KD...-.EA---......T.M.LN-..	..VL......IS.	-------------	-------------	-------------	
Degu	..QQ.P.......Q.PT.TPA-G.D.PP.GGP...M.PRQ-...KD...-.EA---......T.F.LN-..	..VL......I..	-------------	-------------	-------------	
Guinea pig	..QQ.P...M.H.Q.PT.TPA-I.D.PQ.AGP...M.PRQ-...KDA..-.EA---HE....TEM.LN-..	..VL......I..	-------------	-------------	-------------	
Squirrel	...Q...S.......PT...A-I....Q.GGP...M.PRQ-....D..P-.AT---E.....TE...T-SK	.LVM...G..V..	-------------	-------------	-------------	
Rabbit	...Q...........PT..PA-I.G..Q.GGP.LR..PAQ-...KD..M-.EI---.E....TE....-..	..AM...G..V.T	-------------	-------------	-------------	
Pika	...P.P.T.M.S...PTV.PA-I.G.PR..GP.H.M.P.Q-...KD..V-.EI---E......E...N-..	..AM...G..V.T	-------------	-------------	-------------	
Cow	...Q.....Q.....PTVV.S-M.NAVQ.GVP....YQ..-P...QA.G-.M.---E......E...T-T.	....M....L.D.	-------------	-------------	-------------	
Sheep	...Q.....Q.....PTVV.S-M.NAVQ.GVP....YQ..-P...QA.G-.M.---E......E...T-T.	...M....L.D.P	-------------	-------------	-------------	
Dolphin	...Q.P...Q.....PTVV.S-V.N.VQ.GIP....YP..-P...QS.G-.MI---.-.....E..S.-T.	...MN.....D..	-------------	-------------	-------------	
Pig	...Q.....Q.....PTVV.S-I.NPVQ.GVP....YQ..-P...QV.G-.M.---.......E...E-A.	...L......D..	-------------	-------------	-------------	
Alpaca	...Q.....Q.....PTVV.A-I.NSVQ.GLP...VYQ..-P...QV.G-.M.---.E.....E...T-A.	...M......DLP	-------------	-------------	-------------	
Horse	...Q.....Q.A...PTIV.SGI.D.IQ.GGH...VYQRQ-P...QS.G-.M.---............-A.	.TEM..PG....Q	.TE....G....Q	.TEM..PG...PQ	LTEMDFPGPQGPQ	+9c-9e
Rhinoceros	...Q.....Q.....PTIV.AVM.N.GQ.GGP...VYQ.R-P...QS.G-.M.---E......E..TE-A.	..EM..PV..D.P	-------------	-------------	-------------	
Dog	...Q.....Q.......IV.D-I.D..Q.RGT...VYQ.Q-P...QT.G-.ML---E...........-A.	...M...E.....	-------------	-------------	-------------	
Panda	...Q.....Q.......IV.N-I.N..P.GGP...VYQ.Q-P...QK.G-.ML---E...........-A.	...M.....E...	-------------	-------------	-------------	
Ferret	..PQ.....Q.......LL.D-G.D..P..GP...AYQ.Q-P...QA.G-.ML---E.....T.....-A.	...M....L....	-------------	-------------	-------------	
Cat	...Q.....Q.....PTIV.A-I.DA.Q.GGPH..VYQ.Q-P...QA.G-.ML---E...........-A.	...M.........	-------------	-------------	-------------	
Shrew	...Q.H...Q....PPT...A-V.NP.Q.GGP...VYHHRQP...QA.G-.M.---...I....M..N-E.	G..T...E....G	-------------	-------------	-------------	
Star-nosed mole	T..Q.....Q....LPTV..A-I.N.VQ.GGP...VYQ..-P...QA.E-.MI---........V...-AK	..E...PEL.A..	-------------	-------------	-------------	
Hedgehog	...QSH...Q...HLPTVE.A-I.N.IQ.GGP.L.VYQ..-P...QA.G-.MI---........T...EA.	..RM......D..	.LR...A...D..	..QM......ITS	-------------	
Megabat	...Q...T.Q...F.PTVV.A-M....Q.GGP...VYQ.Q-P...QA.G-.MI---E.........S.-A.	...M...G...S.	-------------	-------------	-------------	
Big brown bat	...Q.....Q...H.PSVI.A-I.D.VQ.GGP...VYH.Q-P...QA.G-.MI---.......E....-TK	Q..M.........	-------------	-------------	-------------	
Microbat	...Q.....Q...H.PSVI.A-I.D.VQ.GGP...VYH.Q-P...QA.G-.MI---.......E....-A.	...M.........	......A......	-------------	-------------	
Tenrec	Q..Q...S.P...PVPTD..A-I.DP.Q.AGP.......F-P...QA.S-.AI---L......E....-..	...F...G..S..	-------------	-------------	-------------	
Golden mole	Q..Q.....Q.....PTP..A-I.G.IQNGGP.....Q.Q-P...QS.G-.V.---.......EM...-..	...M...G.....	-------------	-------------	-------------	
Elephant shrew	Q..Q.....Q..S.-PT...V-L.G..P.IGP.....Q.L-P...QSDL-.M.---..........L.-A.	..EMN.GG....I	..EIN.AG..D.T	..EI...D...P.	LPEINFADPQVPT	
Hyrax	Q..Q.....Q.F..L.T.P.A-MHDK.Q.TL...AL.Q.P-P...QKDS-.MI---E...........-..	..EMN....HS.P	-------------	-------------	-------------	
Elephant	Q..Q.....Q.L.P.PTVP.A-I.N..Q.SL...AF.Q.Q-....QN.S-.MI---............-..	..RM...G..S.P	-------------	-------------	-------------	
Manatee	Q..Q....RQ.L.PHPT.P.A-I.G..Q.SL...A.YQ.Q-P...QN.S-.MI---L...........-..	...M........P	-------------	-------------	-------------	
Opossum	QT.Q..KS.Q.S....--SPA-P-DA.QHGVPL..LNQEQ-SHM-QP.DKGAG---..PGL.LGNAGR-..	..EM...GQL.Q.	-------------	-------------	-------------	
Tasmanian devil	Q---..K..Q......--SPP-P-D..QHG.H...L.Q.Q-IHMH-P.DRAMG---..PGG.LGRAGR-..	..ERN..GHL.H.	-------------	-------------	-------------	
Wallaby	Q---..K.VQ.T...P--SPA-P-DATQHG.P...PNQ.QNPG.--P.DR.VG---..P.G.LGRAGR-..	..EL...RQI.H.	-------------	-------------	-------------	
Platypus	RT.Q....KQNA..PPTV.PSAQGPP.QNGDP....LHLQ-P...QE.-K.S.---.E....PKR...-..	..AL..TVQLRQ.	-------------	-------------	-------------	

```
                  exon 10                      exon 11           exon 12            exon 13                                                                              373
Human             IF-QIA-RLISHGPM--PQNKQ--SP | LYPGMLYVPFGANQL | NAP-ARLGIMSSEEVA | GGREDPMAYGAMFPGFGGMRPGFEGMPH--NPAMGGDFTLEFDSPVAA-TKG---PENEEGG------AQG
Chimpanzee        ..-..-........--.....--.. | ............... | ...-............ | ............................--.................-...---.......------...
Gorilla           ..-..-........--.....--.. | ............... | ...-............ | ............................--.................-...---.......------...
Orangutan         ..-..-........--.....--.. | ............... | ...-............ | ...G...........E..K.........--.................-...---.......------...
Gibbon            ..-...-....R..--.....--.. | ............... | ...-...N........ | ...G.....EV..........S......--K................-...---.......------...
Baboon            ..-...-....R..V--.....--.. | .....F.M....... | ...-..........M. | ...G.......I........SSLG....--K................-...---..KG..------...
Macaque           ..-...-....R..--......--.. | .....F.M....... | ...-..........M. | ...G.....E.I........SSLG....--K................-...---..KG..------...
Squirrel monkey   ..-...-....R..--......--.. | ..S..F......... | ...-..........M. | ...GG..G.............S.G....--........L..........-..S---..KV..------...
Marmoset          ..-...-....R..--.......--.L | .....F......... | ...-..........M. | ...GG..G...L.S.......S.G....--.................-...---..KG..------...
Aye-aye           ..-...-...AR..--.......--.. | .....F.LSY..... | ...-G.........M. | .E.GG..T..........I.SSLGEL..--...K..........LGT-...---..KG..------TKD
Bushbaby          ..-...-...AR..--.......--.. | .....F..SY..... | ...-G.........M. | ...GG..G.E.V......L..SLG.L..--................L..-...---..KG..------.PD
Mouse lemur       ..-...-...AR...--T.....--.. | .....F.MSY..... | ...-G.........M. | ...GG............A...S.G.L..--..G.............L..A...---..KG..------..D
Tarsier           V.-...-....Q..V--.....P--.. | .F...F.MSY..... | ...--.........M. | ...GG.L..............RLG....--.................-...---..RG..------..D
Tree shrew        ..-...-....R..--.......--.Q | I....F.MSY..... | ...-..........M. | ...GG................SLG....--.................-...---..KG..------V..
Kangaroo rat      V.-HL.-....RA.I--.D..P--.T | .....F.MSY.T... | .T.-.....F....MP | .A.G-..........E.L.HTLR.LGP--.T.K..........SV-...TKG..KG.EE------P..
Rat               V.-....-HSL.R...--AH..V--PT | F....F.MSY..... | ...-...I.F....MP | .E.GS.....TL...Y..F.QTLR.LNQ--.SPK.....V.V....SV-...---..KG..P------E.
Mouse             V.-....-.S..R...--AH..A--.A | F....F.MSY..... | ...-...I.F....MP | .E.GS.....TL..R...F.QTLRRLNQ--.SPK.....V.V....SV-...---..KG..P------E.
Chinese hamster   V.-....-HSV.R...--AH..A--PS | F....F.MSY..... | ...-...V.F....MP | ...GN.....TL......F.QTLR.LNP--.SPK.....V.V....SV-...---..KG..P------E.
Jerboa            V.-....-HV..R...--A.....--PA | F....F.MSY..... | ...-.....F....MP | .E.GG..G..S....YE.L.HTLR.LRP--.T.K............S.-...---..KG..------EK.
Naked mole rat    V.-....-.F..RV.V--.....P--.T | .....F.MSY.T... | S..A....F....MP | ...GG..T...LL...T...HTIRR..Q--D.T.A..........-...---..KG..A------..
Chinchilla        V.-....-.FVPRVLV--.....P--PT | .......MSY.T... | S..A....F....MP | ...AS.VT...LL...A.V.HTVRR..Q--D.T.A..........-...---..KG..------T..
Degu              V.-....-.FM.RV.V--.....P--PT | .......MSY.T... | ...A....F....MP | ...GS.VT...LL...A.V.HSVRR..Q--D.T.A..........-...---..KG..------...
Guinea pig        V.-....-.FV.RV.V--.....P--AM | .....V.MSY.T... | S..A....F....MP | ...VA.VT..GLL..LT.V.HTIRRV.Q--D.T.A........T.-...---..KG..------...
Squirrel          L.-H..-..V..R..L--.....P--.A | .....F.TSY..... | ...-T...M.....D | ...GG..G..........L.HSLRR.SP--...............-.G-...---..KG..E------.A
Rabbit            V.-......PR..L--....RH--AA | .....F.MSY.G... | ...-......L...MP | .E.GG.........R...I.HSLG....--.................-...---..KG..A------ED
Pika              V.-......PR..L--....RH--TA | .....F.MSY....M | ...-V.........MP | .EGGG......I..R...IKHSLGE..R--.L.WV.ILP....T.G..-I..---..KG..------TPD
Cow               M.-P..-H...R...--.....P--.. | ....IF..TY..... | ...-..........MG | ...G..L....I.........RLG....--..D...............-...---..KG..------..D
Sheep             M.-P..-H...R...--.....P--.. | ....IF..TY..... | ...-..........MG | ...GG.V....I.........SLG...Q--..D.A.............-...---..KG..------..D
Dolphin           M.-P..-.....L..V--.....P--P. | ....IF.MSY..... | ...-..........M. | ..GGG.................LG...P--...............A.G-...---..KG..------..D
Pig               M.-P..-.....Q..V--..D.P--.. | .....F.MSY..... | .S.-.....L....M. | ...GG.L..............NLG...P--.S.K............A.G-...---..KG..------.E.
Alpaca            MLVP.S-..L.R...--.....P--.. | .....F.LSY..... | S.-.....L....M. | ...GG...............SNLG...T--...............-.G-...---..KG..------...
Horse             M.-P..-....AR...--.P....--A. | ....LF.LTY..... | .T.-G.........M. | ...GG...............SSLR...P--.................-...---..KG..------P..
Rhinoceros        M.-P..-..MTQ...--........--.S | ....LF.MSY..... | ...-G.........M. | ...GG................SLG...P--..G............T.-...---..KG..------P..
Dog               V.-...-....R...--.....P--.. | ....IF.MSY..... | ...-G.........M. | ...GS................NLG...--..G.............-.G-...---..KG..------...
Panda             V.-...-....Q...--.....P--.. | ....IF.MSY..... | DT.-G.........M. | ...GG................KLG....--...............-.G-...---..KG..------...
Ferret            V.-...-....R...--.....P--.S | ....IF.MSY..... | ...-..........M. | ...GS.......I.........HLG....--...............-.G-...---..KG..------...
Cat               V.-...-....R...--.....P--.. | ....IF.MSY..... | ...-..........M. | ...GG................NLG.I..--............T...G-...---..KG..------T..
Shrew             L.-H..-....RE.L--S...P--.S | ....IY.MSY..... | ...-G.I.F....MP | ...AS..T..S.YSRL..L..NLA...QNQ...K..........T..G.-...---..KG..------P.R
Star-nosed mole   L.-P..-....AQ..V--.....P--.S | .....FFMSY..... | ...-V.........M. | ...GG.....S..........SLG....--...............T...-...---..KG..------...
Hedgehog          L.-P..-....AR..IAV....P--.S | .F...F.MSY..... | ...-G.........MP | ....G.T.........R....N.GR.HQ--.SPK.....V...T.AV--...---Q.KG...KEGEGDPHH
Megabat           ..-P..-....AQ.T.--.....P--.Q | .....F.MSY..... | .T.-..........M. | ...GG....E.........S.SLG....--H.G............T...G-...---..KG..D------...
Big brown bat     ..-S..-.....Q..L--A...P--.S | .....F.MSY..... | ...-TT........M. | ...GG.......I........SLG...P--.LGK...............-...---..KG..P------V..
Microbat          ..-S..-.....Q..L--A...P--.S | .....F.MSY..... | ...-T.........M. | ...GG.......I.....S...SLG...P--.LGK..............-...---..KG..V------V..
Tenrec            ..-...-.....Q..V--..P...SQ.. | .....F.MSY..... | ...-G.........M. | ...GG............E.I....G..QP--.QGK..........T.F.--...---..KG..------.H.
Golden mole       ..-...-.....Q..V--.......--.. | .....F.M.Y..... | ...-..........M. | ...GG...F.S.....E.......G...P--.QGK...............-...---..KG..------...
Elephant shrew    ML-...-.....Q..V--T.T....--.. | .....F.M.Y..... | ...-G.........MV | ...GG.......I.....RL....G..AP--.QGK..........T.-G.-...---..KG..------T.E
Hyrax             ..-...-.....Q..L--.....E--.. | .....F.MSY.T... | ...-..........M. | ...GG.L............I.SS.G..RP--.HG............N.A..-...---..KG..------VH.
Elephant          M.-...-.....Q.SV--.......--.. | .....F.MSY..... | ...-...........I. | ...GG........V.......L.SS.GR..T--.Q...............PG-...---..KGG..------.HS
Manatee           ..-...-....R..V--.......--.. | ....E.F.MSY..... | ...-.....F....M. | ....G.T....V.........S..G....--.Q............L.........---..KG..------...
Opossum           M.-PMG-....Q..E---.-.AEKA. | .D...F.M.Y...R. | ...-...F.LL....MM | ...GT..G.......---.KS..G...P--............H.Q.TGG-N.S---.GVA..------..D
Tasmanian devil   M.-PMG-...AE..T---.-.TEKA. | ...R.V.M.Y..... | ...-G.F.ML....MM | ...GS..G.......---.KS..GE.TP--...T.........H.L.TGN-N.T---.GAA..------...
Wallaby           M.-PMG-...AEA.T---.-.TEKA. | ...R.F....E..E. | ???????????????? | ...GG..G..S...E---.KS.L..IAP--..T..........R.L.KG.-R.S---.GPA.D.------D.T
Platypus          ..-P..QK...Q..R---.-.AEQ.. | ...ELV.........Q | ..AP....KV....MP | ..GGE.L...SI.....S.....GA.QI--...L.K.....D...TV.-G.P---.GQGGEV------LGS
```

```
                                                                        exon 13                                      500
Human            SPMPEANPDNLENPAFLTELE-PAP--HAGLLALPKDDIPGLPRSPSGKMKGLPS-VTPAAADPLMTPELADVYRTYDADMTTSVDFQEEATMDTTMAPNSLQTSM-PGNKAQEPEMMHDAWHFQEP* (447)
Chimpanzee       .....................-...--............................-..................................................-..................... (447)
Gorilla          ..........P..........-...--............................-..................................................-..................... (421)
Orangutan        ..........P..........-...--.........................H..-......................G...........................-..................... (434)
Gibbon           ..VL......P.........-..A--...........V.S...........F..-I..............NI................D................-..................... (447)
Baboon           .........DP......A...-.VA--...........V.S..............-....G...........................D.V...............-...R.......Y......... (473)
Macaque          .........DP..........-.VA--...........V.S..............-....G...........................D.V..............I-...R.......Y......... (473)
Squirrel monkey  ..........P....VFPD..-..A--...........VSS........R..AL.-I.................G..G.......M....................-.............T....... (446)
Marmoset         ..........P...GVFPD..-..A--...........VSS........R..ALG-F.......M.........G..G............T...............-S.................... (446)
Aye-aye          ..V..DD.A.P....R....L-AGA--RT...TF.E..D.S.A.G.A.QR.RP..-................I.E..G.....PLEATMDT..-.---.DPQEI..-L.....QSQIV.......K.. (417)
Bushbaby         T.G...S.AHP.Q..LPP..A-.GA--.P....F.D.ED.S.A.G.A.QRL.P.G-................I.G..A..V..PL----...T...G..DL.E..SA...R..QAQAAREV.RY.... (418)
Mouse lemur      ..IL....A.P....L.P..A-.DA--.T....F.E.ID.S.A.G.A.QLM.P..-..................E..G.....PL----..VT....T.DP.E..V-..D...Q.QIV.......... (418)
Tarsier          ...L....AHP..Q.LIP..G-.G.--......FS..NV.S.A.G.A.QR..P.R-.....T..........FG..G.....PLS....TI...ST..DTQ...T-.E..V.QS.N........... (423)
Tree shrew       ...L....A.P...EL.P.VV-.GA--.............S.TKGST.QRM.PSG-...............D.G..GV.V..PMGL..........T.D.Q....-..I.....QV........... (421)
Kangaroo rat     ..LH.G..---.G..R.SHIA-SGA--.....GF.D.N..S.A.G.A.QRP.S.G-....NT........SE..DV.GS.V..PLGE-G........S.DTQ..LI-....MHH.Q.V....R..... (424)
Rat              ..LQ..S..KG....L.SQMA-.GA--......F.N.H..NMA.G.A.QR--.LG-.........I.....E..E..G..V..PLGD-G.....I..S.DTQ.PP.-....VHQ.Q-V.N..R..... (422)
Mouse            ..LQ....GKR....L.SQMA-.GA--......F.N.H..SMA.G.A.QR--.LG-.........I.....E..E..G..V..PLGD-G.....I..S.DTQ.PLL-....VHQ.Q-V.N..R..... (422)
Chinese hamster  ..LQ....A.R....L.SQMA-.GA--......F.N.H..S.A.G...QKQ..LG-...............E..G..A..PLGD-G...V.I..S.DTQ.PLV-....VHQSQ-V.N..R..... (425)
Jerboa           ..LE.T.QE.P..Q.L.SHIA-.GA--.....GF.N.N..S.A.G.A.QR....G-..............E..D..G.E...PLSD-G...TE..AS.DTQ..W.-....VHQ.QLV.K........ (426)
Naked mole rat   ..LQ......P..A.L.SQIA-.GA--.E...GF.N.NV.SMA.G.A.QR.RPLG-....TT....I....EA.D..G..V..PL---G....E...T.DTP.SL.-.....HH.QII.N..RY.... (421)
Chinchilla       ..LH.TKAEDA..A.L.SQIA-.GA--RT...GF.N.NV.SMA.G...QR.RPLE-...VT..........EI.E..G..V..PL---G....E...T.DTP..LV-.....RH.Q....P.R..... (422)
Degu             ..LH.TKAEDN.KAGL.SQIA-.GA--R....GF.N.NV.SMA.G...QR.RPLG-....T..........EI.E..G..V..PL---G....E...T.DTP..LA-.....RQAQ....P.R..... (422)
Guinea pig       ..LH.TKGEDP..A.L.SQIA-.GA--.T..FGF.N.NV.SMA.G...HRNRPLE-...VT....I.....EI.E..GG.V..PL---G....E...T.DTP..P.-....VRQ.Q.V.EP....... (422)
Squirrel         ...Q....A.PK...L.SQIA-.GA--......F.N.N..N.A.G.ARQK..PLG-...S............E..E..GD.V..PL.T-G........T.DTQ....-....VHQ.Q.V....R.H... (423)
Rabbit           A.......S.P....L.Q.VA-HGA--......F...N...MA.G.A..KM.V.R-...SD....A.....E..DI.GP.V..PLGT...T.T....T.DTQE..I-....D.HSQ...N..R..... (421)
Pika             .....T..S.PA..GL.Q.IA-HGA--......F...HV.S.AMG.A.QRM.P.R-...SP.G..AN..V.E..DM.GP....PLG....S.T..P.T..TQEIP.-L...D.Q.QI.R.V.R.P... (422)
Cow              ..V...HLADP.S..L.S..A-.GA--LE....N.EGN..N.A.G.A.RSR.FLRG...........G..E..E..G..E..TLGL...T.V.S.AT.DTQH.L.-.R....Q.QIK.......... (422)
Sheep            ..V...HLADP.S..L.S..A-.GA--LG....N.EGN..N.A.G.A.RSR.FLRG...........G..E..E..G..E..TLGL...T...S.AT.DTQHPL.-.R....Q.QIK.......... (422)
Dolphin          P.V.....AD.....L.S..A-.GA--LG....N..GN..N.A.G.A.QSR.PLR-...........G.....E..S..E..TLGL...T.V.S.VT.D.QH...-.......Q.QIK.......... (420)
Pig              ..VA...TADP.S..LFS.VA-SGV--LG....N..GK..N.A.G.A.RSR.P.G-....D.......G...A.E..G..E..TLGL...M...S.AT.Y.EH...-.......Q.QIKR...R..... (421)
Alpaca           ..V..T...DP....L.S.VA-.GA--LG....SA..NV.N.A.-----IR.PFR-...........G.....E..GV.E..TMGL...T.V.S.AT.D......-S.....Q.QNKY.......... (417)
Horse            ..AK..DEA.P..Q.L.S.IA-SG.--LG.......GSV.N.A.G.A.QSR.P.R-...........G..EA.E..G..V..PL..E..T.T...VT.E.P....-....E.EQ.Q.V....R..... (499)
Rhinoceros       ...T..D.A.P....L.S.IA-SG.--LG......QGSV.R.A.G.A.HSR.P.R-...........G...A.E..A.....PLGLE..S.T...VTSE.P....-......EQ.QLVR...R.P... (422)
Dog              ....DV..A.P....L....A-.GA--LG....H...ND.S.A.G.A.QSG.P.R-....D..........I.E..G..V..PL---..TPT...VI.DTQ..L.-.E....Q.QI...G........ (418)
Panda            ..T.DGD.A......DL.PGVA-.GA--LG....H.E..E...A.G.A.QSG.P.R-....D......G...I.E..G..V..PL---..TPT...AV.DTQ..L.-.ES....Q.QI...V...K... (419)
Ferret           ..LT.LD.A.P.S..L.P.GA-.GA--LG....H..GND.NMA.G.A.QSG.PLR-....D......G...I.EN.G..V..PL---..TPT...AS.DTQ..L.-.E.N..Q.QV...V.R..... (418)
Cat              ..R.DV..ADP....L.P.VA-.GA--LG....H..GND.S.A.G.A.QSG.P.R-....E......G...I.E..G..V..PL---..TPT...AI.DTQ....-.E....Q.QI...V........ (418)
Shrew            ..Q...QRADP.ETETRDF.PEA.LGPLG....S...H..SQA.G.A.QNW.F.A-..AD.T.A..N.GI..I.E..G.....PPGL.G.S.T......DAKH.PK-A.S....K.QIS.....R.P... (427)
Star-nosed mole  ........V.P....L.S.VA-LGA--LG....S..GSV.S.VKG.A.QSW.T.K-...........G...A.E..G.....PLGL...S.T.A.VS.DTQ...L-.V......QIT........... (421)
Hedgehog         .RL..R.RLSP....LFQ.AAALEA--LG.F..S....N..S.AMG.A.LIREPSK-I..E.........G.....E..G.....PLNL.G.S.T.S.TTMDIQE..T-....EEQ.QV.....R..... (445)
Megabat          F.T...H.A.P....LPP.GV-.SA--LR.......GSVS..A.G.A.QRV...K-....T..........A.EN.G..T..PL---G.T......VT.DTQ....-.......Q.---..V........ (405)
Big brown bat    ..V....LA.P....L.P.VVM.GA--L.........N..S.V.G.EEHN..S.R-...........G.....EP.A..T..PL---G.T.L....VT.DTE....-.....V.Q.HI..NG....... (409)
Microbat         ..V....LA.P....L.P.V.M.SA--LG.......GN..S.A.G.A.HS..S.R-...........G...G.EP.A..T..PL---G.T.L....VT.DTE....-.....V.Q.HI..NG....... (422)
Tenrec           A.......A.A....L.S.VA-.GA--.GA...F..GLF.S.A.G.A.QSR.PLR-...EV...MV..G.....G..G.....PLGL.G.G.T.P..T.DVS...A-Q.....EQ.PN..KG....... (422)
Golden mole      ..L..T.LA.P....L.L.VS-.GA--.G....F..GN..S.A.G.A.QSR.HFG-.........V..G...A.E..G..V..PLGL.G.T......T.DTQ....-Q.....Q.QI..N..P..... (421)
Elephant shrew   PHR.AG..A......TL.T.FA-.GG--......P..GN.F.VAKG.A.HSW.SLR-....--...A..G...L.E..GP.A..PPG..D.T.T.S.VT.DTA....-.V..G.Q.QI..N......... (457)
Hyrax            .....V..A.P....L...VA-TGA--.G........N.LN.AKG.A.QSS.P.R-...TTT...V..G.D.A.E..A..L..PLNL..GS.T.I.AT.DTQE...-....RG.Q.Q............ (417)
Elephant         .A...T..A.P....L..DVA-TGA--.G....F.NGN.AS.A.G.A.QSR...K-.....N...V..G.T.A.G..T.....PLGL.....T...AT.DTM....-.......Q.QI........... (411)
Manatee          ........A.P....L..DVA-AGT--.G....F..GN..S.A.G.A.QSR.P.R-....T....V..G..EI.G..S.....PLGL.....T...AT.DTQ....-.......Q.QV........... (421)
Opossum          ..G.KG..G...K....P.EI-.WA--QG.I.SF..GR.SS.GGG.A.QV..FLR-G.SPTPESGI..GPS.ITE.FGT.I..PLGML..GPL.I.IE.DTHH..V-Q..ES.Q.QI.Q.V....... (408)
Tasmanian devil  ..VA....E..D..D..P.DF-.WV--QG.I.NF..GRMSK.G.G.A.QI.RPLK-G..PTSE.G..LGT..FTD.FGT.V..PLANL..GPL.V.VE.DTHH..V-Q.TGVRQSQI.E.V....... (405)
Wallaby          ..T..DD.E.I......P.EI-.WA--QG.I.SF..GS.SN.GGGSA.QI.RPLK-G..PTSE.G..LGPS.TTE.FGT.I..PLAMIG.GPL.I.VE.DTHH..V-E..E.HQ.QI.G.V....... (406)
Platypus         PRDGAIPAGHP-..GL.P.RISRLRS-G.A.-TF.S.F..HRGQG.A.QS.LP.G-...PP.......GPS.PLEFFEG.G..PLGL.R..LL....T.DTQS.Y.-...E..QMQVTQ.Q.Q..... (411)
```
