# Supplementary material for: Evolutionary analysis of selective constraints identifies ameloblastin (AMBN) as a potential candidate for amelogenesis imperfecta
Source: BMC Evol Biol. 2015 Jul 30;15:148. doi: 10.1186/s12862-015-0431-0 (PMC4518657; doi:10.1186/s12862-015-0431-0)
Supplement: Additional file 3: — Comparison of intron/exon boundaries and UTR of twelve mammalian AMBN sequences. For scientific names and references, see Additional file 1. (//): sequence not shown; (?): unknown nucleotide; (.): nucleotide identical to human AMBN nucleotide; (−): indel. (PDF 143 kb) [file 12862_2015_431_MOESM3_ESM.pdf]

Additional file 3. Comparison of intron/exon boundaries and UTR of twelve mammalian *AMBN* sequences.
For scientific names and references, see Additional file 1 1. (//): sequence not shown; (?): unknown nucleotide; (.): nucleotide identical to human *AMBN* nucleotide; (-): indel.

**Species**	**5′UTR**	**Exon 1**	**5′ Intron 1 3′**	**Exon 2**	**5′ Intron 2**
Human	caggccctgagagcacagtgc	ATG // AAG	**gt**aaaatgggatttatgatt // tccaacttaattatgtttt**ag**	AAT // CCG	**gt**aagtcagtctt-tagagtg
Tarsier	....	ATG // AAG	........-........... // ......cc......a.g....	ATT // CCG	............c.......
Tree shrew	?????????????????????	??? // ???	????????????????????? // .t...tcc.t.....c.....	ATT // CCG	.......ga....c.c..a..
Mouse	....-....g........t.a	ATG // AAG	........t........c... // .a....cc..c.g........	ATT // CCG	.......ca..cct..cccg.
Rabbit	g...t..............aa	ATG // AAG	........t............ // .t....cc............	ATT // CCG	............gc.g.tca.
Cow	a....aatg............	ATG // AAG	........t............ // .....tcc..c.c.....c..	ATC // CCG	...........c.c.g.g.gt
Horse	....	ATG // AAG	........t............ // ct..c.cc..c.c.....c..	ATC // CCG	.........ag.ct.ct..ac
Dog	...........aa........	ATG // AAG	........t............ // ......c.....c.....c..	ATC // CCG	.........ag..t.gt..at
Microbat	actat.t..gtt.gcatcac.	AGG // AAG	........t............ // ......c.g.c.c.....c..	ATC // CCG	.......ccagcag-...cac
Elephant	tcc.gg.c..a.....g....	CTG // CAG	......c.t....ct.c.... // ......cc....c........	ATC // CCG	.........ag.at.cc..a.
Tasmanian d	....tt...g..a..ag..a.	AAG // AAG	......aaatggg..t..t.a // ct....c.....c.c..cc..	AAA // CAG	......tg..tcac.ttgagc
Platypus	.....t...ga.a...g....	AAG // AAG	........tat.c.gt..gga // atg.c.catt..c.....c..	GTC // CCG	....a..t.at.ct.gctact
	**Intron 2 3′**	**Exon 3**	**5′ Intron 3 3′**	**Exon 4**	**5′ Intron 4**
Human	attattcttattttcattc**ag**	TTC // GAG	**gt**atgtattgtcagaattttt // cccacttttttttcttgat**ag**	ACA // CAG	**gt**aatca-tatt-tct--tat
Tarsier	..g..........c...gt..	GTG // GAG	........g..t...g..g.. // taac.ac......t..a....	ACA // CAG	.....t.-...gt..aaa...
Tree shrew	.c...........c....t..	ATG // GAG	......t..a.tt..g.g... // .aatt..........ca.c..	ACA // CAG	.......-gt..aaaa--...
Mouse	..c....a.....ct...t..	GCA // GAG	........ca.....ggcg.g // tta.cc.ac.......t....	ACA // CAG	....ct.-..cgaga.--g.a
Rabbit	.........g...c....t..	GCA // GAG	..........c......c... // a..ta...........t.c..	ACA // CAG	.......-...atc.agag..
Cow	.............c....t..	GCG // GAG	...............g.a... // gagtt..c............	ACA // CAG	......g-....t..aaa...
Horse	.............c....t..	GCG // GAG	...............g.a... // .....-.......tc.t....	ACA // CAG	....c.t-....t..aaa...
Dog	.............c.t.ct..	GTG // GAG	...g...c.......g.a... // a.ttt......c.t..a.c..	ACA // CAG	....c..-..g.t..aaa...
Microbat	.............c.g.gt..	GTG // GAG	...c...........gcac.g // ..act...........t.c..	ACA // CAG	....a.t-.g..t..aaa...
Elephant	..g..........c....t..	GTG // GAG	......t.gcat.t...c..g // .....-..........t....	ACA // CAG	....ct.c....t..aaa.t.
Tasmanian d	..g.c.t..g.c.cat.....	GTA // GAG	....a....t.t...g...ac // aa.tc..c..c.c...a....	ACA // CAG	....ctgt.t..aaa.gttcc
Platypus	tg.t.ct..g...c.ttgt..	GTC // GAG	....t.t..tattat.a.aa. // aa.c..c.c.cc.....g...	ACA // CAG	.....gtt.c..c.aatg.c.
	**Intron 4 3′**	**Exon 5**	**5′ Intron 5 3′**	**Exon 6**	**5′ Intron 6**
Human	tttttttatccatgtcttt**ag**	TAT // CAG	**gt**gagtgaatagcatcaatat // tcttttcaaatttctctgc**ag**	TAT // GAG	**gt**acttcctttctcttgaagt
Tarsier	.c.....c.............	TAT // CAG	..ag......ta.....g.g. //	TAT // GAG	....catgc...ctc.ctctg
Tree shrew	....g.ct...c.a.......	TAT // CAG	..a...t...t........g. //	TAT // GAG	............c..-....g
Mouse	.gc...ct.tgtctctg.c..	TAT // CAG	..a..ct...t.....c..g. // ....a...	TAT // GAG	...a...t..ggagccc.cc.
Rabbit	cc.....cc..g.........	TAT // CAG	..a...t...t......g.t. //	TAT // GAG	...a....c....-.g.....
Cow	......cc.............	TAT // CAG	..a...t.g.t.......c.. // ..c..................	TAT // GAG	....c.........-.....
Horse	c....cgc.............	TAT // CAG	..a...t..ct........g. // ..c..................	TAT // GAG	...a..t.....c..-.....
Dog	....cccc.g...........	TAT // CAG	..a...t...t.t......g. // ..c..................	TAT // GAG	...ag..tc.caag.ca..t.
Microbat	c....c.cc.....a......	TAT // CAG	..a...t.g.ta.......tg // ..c..................	TAT // AAG	...aa.....c.c..-.....
Elephant	cccccccccc......g.....	TAT // CAG	..ata.t--.g-.......g. // ......t..............	TAT // GAG	...a.gat...c...-..t.g
Tasmanian d	.c.acc.catgtccctc....	TTC // CAG	.....ctcc.tc.t..c..tc // ..ca..ttcc..c..t.....	TAT // GAG	..ca..g.a.ga.t.cctta.
Platypus	cg.g..ctgttgctc...c..	TTC // CAG	.....ctctactttgattcta // c.accatttt.c.g...c...	TAC // GAG	..cac.t.a..a....tt.cg
	**Intron 6 3′**	**Exon 7**	**5′ Intron 7 3′**	**Exon 8**	**5′ Intron 8**
Human	taatatttatctgtgatat**ag**	CTC // TCA	**gt**aagtacagatctcaatgag // taatatttatctacaatat**ag**	CTC // TCA	**gt**aagtacagatctcagtgag
Tarsier	........c............	CTC // TCA	.......t.a......g.... // -	- // -	-
Tree shrew	.............ct.c....	CTA // ACA	..............c...ga.t // -	- // -	-
Mouse	........cctca...c.c..	GTA // ACC	............t.....a.. // -	- // -	-
Rabbit	..............atc....	CTC // ACC	..........c.......... // -	- // -	-
Cow	..............a..gc..	CTA // CCG	..........a........a.. // -	- // -	-
Horse	..................gc..	CTA // CAA	.... // .g..........gtgg.gc..	CTA // CAA	.......t........a....
Dog	....g.........a..gc..	CTA // TCA	.......t....t...g.... // -	- // -	-
Microbat	..c...........g..gc..	CTA // TCC	..........c.......... // ..c.g.......gtg..gc..	CTA // CCC	.................a....
Elephant	....	TTG // CCA	..................act // -	- // -	-
Tasmanian d	a..c......t.a....gc..	CTA // TCG	..........c..t.t....gc // -	- // -	-
Platypus	.g.c....g.t.aca...c..	TTA // TCA	..g.......c.t.t...tgt // -	- // -	-

	Intron 8 3′	Exon 9a	5′ Intron 9 3′	Exon 10	5′ Intron 10
Human	taatatttatctgtaatat**ag**	CTC // ACA	**gt**aagtacagatctcaatgag // ctgttttctacca-tttaa**ag**	ATT // CCA	**gt**aagttttttttt--aa-tac
Tarsier	-	- // -	- // g.a..........t.......	GTG // CCA	.....c.......--..a...
Tree shrew	-	- // -	- // a.t......-g.tac..g...	ATA // CAA	..........c.a--ctt..a
Mouse	-	- // -	- // ...c....c...t-c......	GTG // GCT	......g.c.gc.--ttc...
Rabbit	-	- // -	- // t........-g.tac...c..	GTG // GCA	...........cc--ttt.tt
Cow	-	- // -	- // ..-c........-.......	ATG // CAG	............---..-c..
Horse	..............g..gc..	CTG // CAA	.......t............. // ......c...t..-c......	ATG // CCA	.....g.......tt..-...
Dog	-	- // -	- // ..a.....a....-c..cc..	GTC // CCG	..........aa---..a..t
Microbat	..c.g.........g..gc..	CTA // TCC	.........c........... // ....-c......	ATA // TCA	............---..a...
Elephant	-	- // -	- // .aac.......t.-c......	ATG // CCA	.........a.cacacca.tt
Tasmanian d	-	- // -	- // t.ta..ctgctttc.g.....	ATG // CCA	.........gg.gatggtggt
Platypus	-	- // -	- // ....tc....-.c.g...	ATA // CCA	........c..ccacttcact

	Intron 10 3′	Exon 11	5′ Intron 11 3′	Exon 12	5′ Intron 12
Human	aatgattgta-ttttattt**ag**	CTT // TTG	**gt**aagtccatattctataaaa // gcaatatttctttttgaac**ag**	AAT // GCA	**gt**gagtaatgtcttctaactc
Tarsier	..ct.c.c..-..........	CTT // TTG	.... // c..c.g..............	AAT // GCC	....
Tree shrew	..cc.c....-..........	ATT // TTG	..........c...g..... // .g...gc.............	AAT // GCC	.........a...........
Mouse	..ct...t.g-..c.gc.c..	TTT // TTG	.........c.....g.c... // ...c.g....-..........	AAT // CCT	.......ccac........a.
Rabbit	...t.....-..........	CTT // CTG	.......t......c...... // .t...g..............	AAT // CCC	.................g...
Cow	g..t......t..a.......	CTT // CTG	.......tt...c....ttt. // t..gcg..............	AAC // GGG	...............a.....
Horse	g.ct...a..t..a.......	CTT // TTG	.......tg..gc........ // ...gag........c......	AAC // GCA	.........c......a.....
Dog	g..tg.c...t..a.......	CTT // TTG	.......tg...c...c.g.. // ...c.g..c............	AAC // GCT	...............c....t
Microbat	t..t......t..a.......	CTT // CTG	.......t.....t...g... // ...g.g.........t.....	AAT // GCG	.........a.....a.....
Elephant	g.ct.....ct..a.......	CTT // TTG	......gt.........tt.. // a....g....cc...t.....	AAC // GCG	...............a.....
Tasmanian d	..cc...tc.t.ca.g.c...	CTT // TTG	......a...g..a.c..g.g // ttc..ta..t.c.........	AAT // ATG	..a...g..tc.c..ctc..t
Platypus	...c..t.c.t..c...c...	CTT // CAG	......t...g..a..ag..c // ctgc.tc......c.acc...	AAT // CCG	.......ttagaaatagtatt

	Intron 12 3'	Exon 13	3′ UTR
Human	acgaat-gtttttttttcc**ag**	GGC // TGA	cagctctaaga-tattagcta
Tarsier	.t.g..--.....c...g...	GGA // TGA	tg..c..g...-...c.....
Tree shrew	tttgt.-t.g....g..t...	GGA // TGA	..a.ct.g...-.cac..tc.
Mouse	.t.g.c-.-...g........	GGA // TGA	t.a.ct.g.c.-..gc.....
Rabbit	t.tttg-a.cc..........	GGA // TGA	ag..ct.g...-.c.cc...g
Cow	tg.t.a-a.gg..........	GGA // TGA	g..tctgg...-...cg....
Horse	tg.t.a-a.gg...c......	GGA // TGA	g...ct.gcag-...c.....
Dog	gtc...g..............	GGA // TGA	g.a.ct.....t...c.....
Microbat	tt.gtaaa...c.........	GGA // TGA	g...ct.g.c.-...c.....
Elephant	.t....-.gc........t..	GGG // TGA	a...ct.g...-..g.t..gt
Tasmanian d	tt.tcctc.ga..c...t...	GGA // TGA	a..gct.ga..agtca.t.ac
Platypus	cg..t.g.ggg..c.ctg...	GGA // TGA	gcttg..cg..ccgagca..g
