# Supplementary figures and images for: Evolutionary analysis of selective constraints identifies ameloblastin (AMBN) as a potential candidate for amelogenesis imperfecta
Source: BMC Evol Biol. 2015 Jul 30;15:148. doi: 10.1186/s12862-015-0431-0 (PMC4518657; doi:10.1186/s12862-015-0431-0)

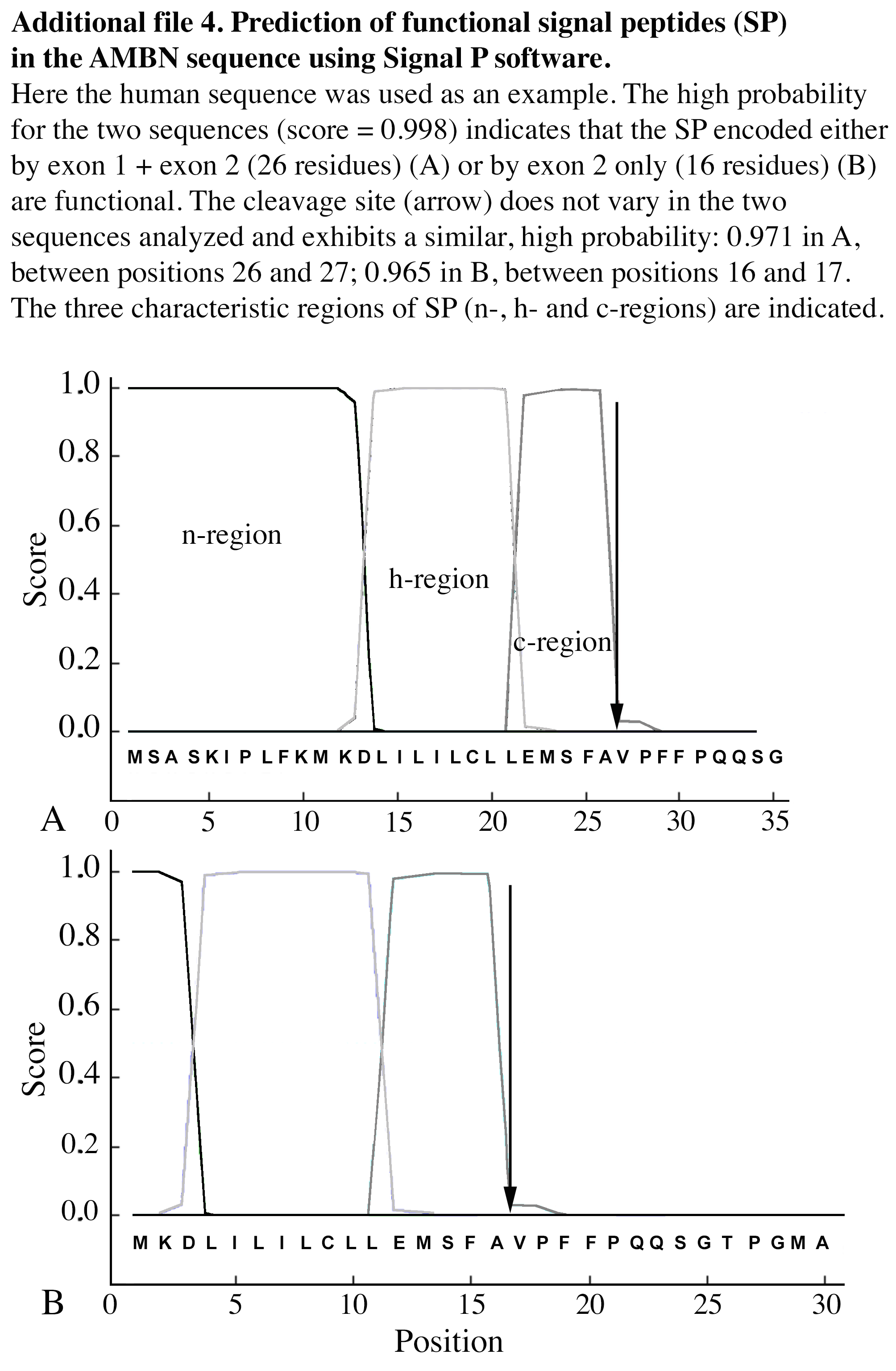

Supplement: Additional file 4: — Prediction of functional signal peptides (SP) in the AMBN sequence using Signal P software. Here the human sequence was used as an example. The high probability for the two sequences (score = 0.998) indicates that the SP encoded either by exon 1 + exon 2 (26 residues) (A) or by exon 2 only (16 residues) (B) are functional. The cleavage site (arrow) does not vary in the two sequences analyzed and exhibits a similar, high probability: 0.971 in A, between positions 26 and 27; 0.965 in B, between positions 16 and 17. The three characteristic regions of SP (n-, h- and c-regions) are indicated. (TIFF 488 kb) [file 12862_2015_431_MOESM4_ESM.tif]
