# Supplementary material for: Evolutionary analysis of selective constraints identifies ameloblastin (AMBN) as a potential candidate for amelogenesis imperfecta
Source: BMC Evol Biol. 2015 Jul 30;15:148. doi: 10.1186/s12862-015-0431-0 (PMC4518657; doi:10.1186/s12862-015-0431-0)
Supplement: Additional file 5: — Amino acid composition of AMBN. The amino acid percentages are given for the full length sequence and the proline-rich region for six representative mammalian AMBN, the putative ancestral mammalian sequence, and the crocodile, and are compared to average frequency in most proteins. The percentages of proline and glutamine are in bold characters. (PDF 100 kb) [file 12862_2015_431_MOESM5_ESM.pdf]

Additional file 5. Amino acid percentage of AMBN.

The amino acid percentages are given for the full length sequence and the proline-rich region for six representative mammalian AMBN, the putative ancestral mammalian sequence, and the crocodile, and are compared to average frequency in most proteins. The percentages of proline and glutamine are in bold characters.

		**Human**		**Mouse**		**Cow**		**Elephant**		**Opossum**		**Platypus**		**Ancestral**		**Crocodile**	
Amino acids	Average frequency	Total 447 aa	Proline-rich 125 aa	Total 422 aa	Proline-rich 110 aa	Total 419 aa	Proline-rich 110 aa	Total 411 aa	Proline-rich 110 aa	Total 405 aa	Proline-rich 96 aa	Total 411 aa	Proline-rich 100 aa	Total 410 aa	Proline-rich 99 aa	Total 407 aa	Proline-rich 86 aa
Leu(L)	9.0	10.96	12.80	9.95	7.27	10.50	6.36	9.49	8.18	10.12	9.37	10.22	8.00	8.00	6.06	9.58	13.95
Ala(A)	8.3	8.28	6.40	8.53	4.54	7.88	3.64	8.27	5.45	5.68	6.25	6.32	6.00	8.80	7.07	7.62	3.49
Gly(G)	7.2	8.50	5.60	9.48	5.45	10.02	4.54	10.22	2.73	13.83	7.29	10.22	2.00	11.70	4.04	11.06	3.49
Ser(S)	6.9	7.38	7.20	6.87	4.54	6.20	6.36	7.30	7.27	7.16	8.33	6.32	5.00	5.40	4.04	3.93	5.81
Val(V)	6.6	2.46	3.20	4.03	4.54	3.34	6.36	2.92	2.73	1.97	2.08	3.41	4.00	1.70	3.03	4.42	3.49
Glu(E)	6.2	6.04	5.60	5.45	7.27	5.49	5.45	3.65	4.54	5.93	6.25	5.11	6.00	4.90	6.06	4.67	5.81
Thr(T)	5.8	4.47	3.20	4.50	4.54	5.01	4.54	6.57	3.64	4.94	2.08	4.62	3.00	5.40	5.05	5.41	4.65
Lys(K)	5.7	3.36	4.00	3.08	1.81	3.10	2.73	2.68	3.64	3.21	3.12	2.68	4.00	2.70	5.05	0.74	1.16
Arg(R)	5.7	2.46	1.60	3.55	0.90	3.34	1.81	3.16	2.73	2.47	2.08	3.89	4.00	2.40	1.01	3.44	3.49
Asp(D)	5.3	4.70	4.00	2.61	2.73	3.58	2.73	2.92	1.81	3.21	2.08	3.41	2.00	3.40	2.02	2.70	0.00
Ile(I)	5.2	2.01	0.80	2.61	2.73	2.86	0.90	2.43	1.81	2.47	0.00	2.68	1.00	2.90	3.03	2.70	1.16
**Pro(P)**	5.1	**14.54**	**23.20**	**14.69**	**27.27**	**14.32**	**24.54**	**13.14**	**22.73**	**12.84**	**19.79**	**16.54**	**28.00**	**14.10**	**22.22**	**12.53**	**20.93**
Asn(N)	4.4	2.91	0.80	3.32	1.81	2.86	0.90	3.65	1.81	2.96	2.08	2.92	3.00	2.90	0.00	3.68	2.33
**Gln(Q)**	4.0	**6.49**	**11.20**	**7.35**	**11.82**	**7.64**	**15.45**	**9.25**	**18.18**	**8.39**	**16.67**	**9.73**	**16.00**	**9.80**	**20.20**	**11.79**	**20.93**
Phe(F)	3.9	4.47	2.40	4.26	3.64	3.82	3.64	4.14	3.64	4.44	2.08	5.11	1.00	4.40	2.02	3.19	1.16
Tyr(Y)	3.2	2.01	1.60	2.03	1.81	1.91	0.90	2.19	1.81	1.73	2.08	1.70	3.00	2.20	3.03	2.95	2.33
Met(M)	2.4	5.59	1.60	4.26	1.81	4.77	4.54	4.87	2.73	4.94	2.08	2.68	0.00	5,60	1.01	4.91	1.16
His(H)	2.2	2.46	4.00	2.61	4.54	2.39	3.64	2.19	3.64	2.47	5.21	1.70	3.00	2.40	4.04	3.44	3.49
Cys(C)	1.7	0.22	0.00	0.00	0.00	0.24	0.00	0.24	0.00	0.25	0.00	0.00	0.00	0.24	0.00	0.25	0.00
Try(W)	1.3	0.67	0.80	0.71	0.90	0.72	0.90	0.73	0.90	0.99	1.04	0.73	1.00	0.73	1.01	0.98	1.16
